# Linking regional stakeholder scenarios and shared socioeconomic pathways: Quantified West African food and climate futures in a global context

**DOI:** 10.1016/j.gloenvcha.2016.12.002

**Published:** 2017-07

**Authors:** Amanda Palazzo, Joost M. Vervoort, Daniel Mason-D’Croz, Lucas Rutting, Petr Havlík, Shahnila Islam, Jules Bayala, Hugo Valin, Hamé Abdou Kadi Kadi, Philip Thornton, Robert Zougmore

**Affiliations:** a International Institute for Applied Systems Analysis (IIASA), Ecosystems Services and Management Program, Schlossplatz 1, A-2361 Laxenburg, Austria; b Environmental Change Institute (ECI), University of Oxford, South Parks Road, OX1 3QY Oxford, United Kingdom; c CGIAR Research Program on Climate Change, Agriculture and Food Security (CCAFS), University of Copenhagen, Faculty of Science, Department of Plant and Environmental Sciences, Rolighedsvej 21, DK-1958 Frederiksberg C, Denmark; d Copernicus Institute of Sustainable Development, Utrecht University, Heidelberglaan 2, P.O. Box 80.115, 3508TC Utrecht, The Netherlands; e International Food Policy Research Institute (IFPRI), Environment and Production Technology Division,2033 K Street, NW, Washington, DC 20006-1002, USA; f World Agroforestry Centre (ICRAF), West and Central Africa Regional Office − Sahel Node, BP E5118, Bamako, Mali; g Institut National de la Recherche Agronomique du Niger (INRAN), BP 429, Niamey, Niger; h International Crops Research Institute for the Semi-Arid Tropics (ICRISAT), BP 320 Bamako, Mali

**Keywords:** Agriculture, Climate change, Representative agricultural pathways, Shared socioeconomic pathways, Stakeholders, West Africa

## Abstract

The climate change research community’s shared socioeconomic pathways (SSPs) are a set of alternative global development scenarios focused on mitigation of and adaptation to climate change. To use these scenarios as a global context that is relevant for policy guidance at regional and national levels, they have to be connected to an exploration of drivers and challenges informed by regional expertise.

In this paper, we present scenarios for West Africa developed by regional stakeholders and quantified using two global economic models, GLOBIOM and IMPACT, in interaction with stakeholder-generated narratives and scenario trends and SSP assumptions. We present this process as an example of linking comparable scenarios across levels to increase coherence with global contexts, while presenting insights about the future of agriculture and food security under a range of future drivers including climate change.

In these scenarios, strong economic development increases food security and agricultural development. The latter increases crop and livestock productivity leading to an expansion of agricultural area within the region while reducing the land expansion burden elsewhere. In the context of a global economy, West Africa remains a large consumer and producer of a selection of commodities. However, the growth in population coupled with rising incomes leads to increases in the region’s imports. For West Africa, climate change is projected to have negative effects on both crop yields and grassland productivity, and a lack of investment may exacerbate these effects. Linking multi-stakeholder regional scenarios to the global SSPs ensures scenarios that are regionally appropriate and useful for policy development as evidenced in the case study, while allowing for a critical link to global contexts.

## Highlights

• Stakeholder driven regional scenarios for West Africa linked with SSPs.
• Make comparable scenarios coherent across levels through quantification.
• Negative effects of climate change on agriculture exacerbated by low investment.
• Increased productivity can lead to trade-offs between regional and global land use.
• West Africa cannot fully meet the growing demand for food and livestock feed.

## Graphical abstract

**Figure:**
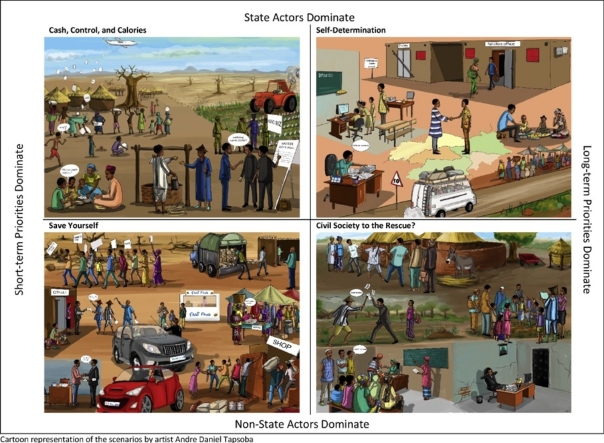

## 1 Introduction

Climate change is a significant source of uncertainty for the food security, health and livelihood of the poor in many of the world’s vulnerable regions, interacting with and compounding other sources of uncertainty such as socioeconomic development, political stability and the effects of widespread ecosystem degradation (IPCC, 2014). Among the most vulnerable regions is West Africa, where 75% of the population of the fifteen countries that are members of the Economic Community of West African States (ECOWAS) live on less than $2 a day and more than 35% of the regional GDP is derived from agricultural production (Hollinger and Staatz, 2015, World Bank, 2011). Though the region is home to currently less than 5% of the world’s people, in the future it may be the fastest growing (Jiang and O’Neill, 2015, Kc and Lutz, 2014) and one of the most exposed to climate change due to its dependence on (rainfed) agriculture, and the estimated negative impacts of climate change (Leclère et al., 2014, Müller et al., 2011, Roudier et al., 2011). Those involved in government policy, private sector investments, civil society action and other strategic processes must consider the interacting uncertainties of development and climate change in an integrated fashion when planning for the future (Vermeulen et al., 2013).

Scenario-guided planning allows decision-makers to engage with uncertain futures and assess and improve the feasibility, flexibility and concreteness of their plans (Vervoort et al., 2014). The international climate change community is developing a set of global scenarios, consisting of various combinations of radiative forcing scenarios (Representative Concentration Pathways or RCPs) and socioeconomic and policy scenarios (Shared Socioeconomic Pathways; SSPs, and Shared Policy Assumptions; SPAs) that when combined can be used to examine the impacts of climate change. These scenarios also provide a global context and/or template for processes at lower geographical levels that seek to use scenarios to guide regional, national or sub-national planning (O’Neill et al., 2014). Conversely, there is scope for sub-global processes to complement the shared socioeconomic pathways (SSPs) with more regional contextualization of assumptions and results, even when using scenarios in the global setting. Regionally specific scenarios serve to assist policy makers in developing robust agriculture and climate adaptation strategies, while also providing the scientific community working at the regional, national, and sub-national level with multiple pathways for development that can be disaggregated or linked to adaptation assessments (Antle et al., 2015, Kihara et al., 2015, Valdivia et al., 2015).

The frameworks to develop the global SSPs have been thoroughly documented (O’Neill et al., 2014, Schweizer and O’Neill, 2013, van Ruijven et al., 2014, van Vuuren et al., 2014), linked to previous scenario assessments (van Vuuren and Carter, 2013), and recently integrated with climate change and quantified (Riahi et al., 2016). They are just beginning to be scrutinized through regional and national (Absar and Preston, 2015), and human impact (Hasegawa et al., 2015) lenses.

In this paper, we present a process in which a set of stakeholder-generated, regional scenarios for West Africa was linked quantitatively to the SSPs by using the regional stakeholder scenarios to critically examine and adapt SSP assumptions made for the region. This way, a set of scenarios was created that focuses principally on regional challenges but has been made coherent with the SSPs (Zurek and Henrichs, 2007), allowing for a global situating of the scenarios. The resulting set of scenarios was designed to be used for planning by policy makers (in the widest sense, including private sector and civil society groups) at national and regional levels and have been used for this purpose in a number of planning processes, among which are national policy guidance processes in Burkina Faso and Ghana. The process was led by the CGIAR Program on Climate Change, Agriculture and Food Security (CCAFS).

We present this process as 1) an example of using global models to quantify regional scenarios to balance the need for regional perspectives with the need for connections to global futures; and 2) to more specifically examine the implications for agriculture and food security in West Africa under future climate and socioeconomic uncertainty.

In this paper we will first describe our participatory scenario development methodology, including how the scenarios were linked across levels and quantified. Then we will present the resulting regional scenarios: the socioeconomic drivers of change and the quantitative modeling results, highlighting the link to the SSPs by their narratives, scenario drivers, and challenges to adaptation. Finally, we will discuss the benefits and drawbacks of our approach of linking regional and global scenarios and compare it to alternative approaches. A note on terminology: following Cash et al. (2006) we use ‘level’ rather than ‘scale’ to describe levels such as ‘regional’ and ‘global’.

## 2 Methodology

### 2.1 Main process objectives and design choices

Scenarios are hypothetical futures expressed through narratives, numbers or other means (visual, interactive), to explore different directions of change (van Notten, 2006, Vervoort et al., 2010). The CCAFS West Africa scenarios provide globally contextualized meso-level futures for policy guidance at regional, national and sub-national levels across West Africa. A number of policy guidance processes were co-developed between the project researchers and policy makers, and designed to directly examine a given policy or plan in the context of multiple scenarios, leading to an assessment and an improvement of the plan’s robustness in the face of future uncertainty, based on new insights coming from the examination of the plan through each different future scenario (Vervoort et al., 2014).

This strong focus on regional and national policy guidance has consequences for how the regional scenarios and global SSPs should be linked. To ensure policy relevance, drivers considered to be the most important at the regional level should frame the scenarios, and policy makers should be involved in the identification of these drivers and the development of the scenarios (Wilkinson and Eidinow, 2008). Multi-level scenario processes can exhibit different degrees of integration of scenarios across levels, though they are often conceived through a top-down process (Biggs et al., 2007, Kok et al., 2007, Kok et al., 2006a, Kok et al., 2006b, Shaw et al., 2009; Kok et al. In Review). Zurek and Henrichs (2007) describe the different possible degrees of linkage between scenarios organized at different geographic levels, from ‘equivalent’ (the scenarios are the same at different levels) to ‘independent’ (unconnected scenarios). We start with regional scenarios that Zurek and Henrichs would categorize as ‘comparable’ to the global SSPs − in that they have a similar scope of concern, but the framing drivers and assumptions of the scenarios are not connected. This comparable regional set of scenarios was then quantified to provide inputs for global modeling, in a process that mapped the regional scenarios to the global SSPs in terms of quantitative drivers. We will argue that this process moved the regional scenarios toward being ‘coherent’ with the global SSPs – meaning that the regional scenarios and the global SSPs map to each other in terms of content and assumptions. Having two different, comparable starting points for the scenarios at each level means that the regional scenarios provide an independent, regionally grounded perspective from which the regional assumptions for the SSPs can be examined and adapted. At the same time, moving the scenarios from comparable toward coherent through the quantification process means that the scenarios can be situated in global SSP contexts − which is essential to understanding the development of West Africa’s future in the face of global drivers of change.

### 2.2 Scenario development and framework

The CCAFS scenarios process in West Africa started by examining, with regional stakeholders, the impacts of future climate and socioeconomic drivers on food security, environment and rural livelihoods. Scenarios were developed over three separate workshops. Regional stakeholders took ownership of the process by offering information on the relevant drivers of change as they related to agriculture, food security and climate adaptation/mitigation in the future of West Africa (in Workshop 1), creating narratives of each scenario (in Workshop 2) and providing semi-quantitative estimates for scenario variables and model inputs, in close collaboration with the modeling teams (in Workshop 3). Although Mali, Niger, Burkina Faso, Senegal and Ghana are the focus countries for the CCAFS West Africa program, participants were also included from regional and international organizations to provide a regional perspective. The regional participatory process depends on having the right balance of participants with diverse interests. To this end we aimed for a mix of participants across focus countries, sectors, disciplines, and gender (for more detail on stakeholder backgrounds see Appendix A in Supplementary materials). Additionally, it was important to select participants who could both offer in-depth expertise and influence strategic processes in their organizations. 94 participants from governments (agriculture and environment ministries, meteorological institutes), research organizations, national and regional civil society organizations (CSOs), international non-governmental organizations (INGOs), academia and the media participated in the original development of the scenarios over the three workshops (Palazzo et al., 2016). The team involved in the selection of the stakeholders included key project partners from the West African Climate Change, Agriculture and Food Security program (CCAFS), from the Agriculture and Rural Development office of the Economic Community of West African States (ECOWAS), and from the West African Council for Agricultural Research and Development (CORAF/WECARD). Together, this organizing team was able to use an extensive regional network for the invitation of participants.

The CCAFS scenarios were created to represent regional developments over time on the way to a 2050 time horizon. Stakeholders outlined four scenarios, structured along two axes of uncertainty, using narrative flowcharts, conceptual maps, storylines, and a range of trend indicators including information on governance, agriculture, food security and livelihoods. Participants selected two axes from a broad set of future uncertainties that were deemed most relevant and uncertain for the region. The axes identified were 1) whether state or non-state actors dominate the regional development process; and 2) whether short or long-term priorities dominate policy-making. Other drivers, such as economic and population growth, were considered important, but less uncertain − which meant that they were selected to play a contributing role in each of the scenarios. Their impacts were different across the scenarios and depended on the other main driver/axes states, but these drivers did not define the scenario. Both drivers defining the scenarios are essentially governance drivers – participants saw regional governance as the most critical and uncertain dimension of West Africa’s future, that could, for instance, determine the direction of investments into development, the use of resources, and other drivers. With ‘non-state actors’, participants meant both private sector and civil society actors – the resulting scenarios where non-state actors play a prominent role resulted in a dynamic interplay between private sector and civil society actors. While each scenario describes the future to 2050, in the scenarios where governance focuses on short-term priorities, this does not mean that the scenarios are themselves shorter. Instead, throughout the time period of these scenarios, short-term concerns are given priority. This results in a relative lack of investment in long-term projects. Thus short cycles of growth and investment make developments in the two scenarios with this characteristic more unstable.

**Fig. 1:**
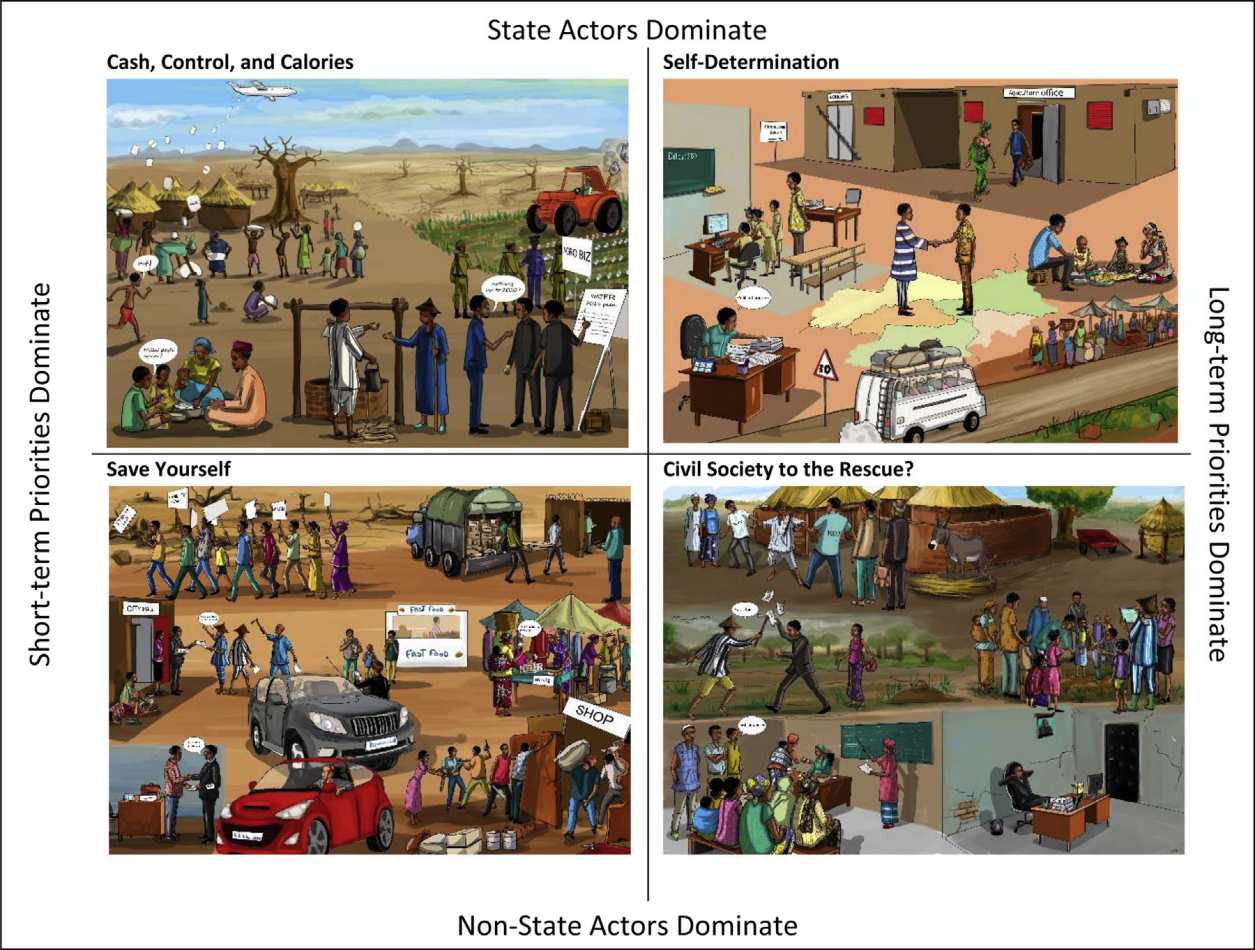
Cartoon representations of the four CCAFS West Africa scenarios along the two axes of uncertainty.

Source: Drawings by artist Andre Daniel Tapsoba

*Self-Determination* is a scenario where state actors dominate development and agendas are focused on the long-term. *Cash, Control, Calories* is a scenario where state actors dominate development and with a short-sighted agenda setting. *Civil Society to the Rescue?* is a scenario with non-state actors, such as the private sector and CSOs, dominating regional development with a long-term strategic agenda. *Save Yourself* is a scenario where non-state actors dominate the regional development and their focus is on the short-term. Cartoon representations of the CCAFS scenarios (based on the scenario narratives) shown along the two axes are presented in Fig. 1. Narrative summaries of the scenarios and details of the development process are found in Appendix A in Supplementary materials.

### 2.3 Quantification of the CCAFS scenarios

Following the development of the qualitative scenarios, stakeholders provided a detailed look into the scenarios by signaling the logic of change and magnitude of change (given as +,=, and −) for a set of indicators that represent the scope of interest for the future of food security, livelihoods, and environments (A full list of indicators appears in Appendix Table B1 in Supplementary materials). To fully quantify the scenarios, a subset of these indicators were given numerical values and used as model drivers in two global partial-equilibrium economic models of the agriculture sector– GLOBIOM (Havlik et al., 2014), developed by the International Institute for Applied Systems Analysis (IIASA), and IMPACT (Robinson et al., 2015), developed by the International Food Policy Research Institute (IFPRI).

#### 2.3.1 Quantification of the scenario drivers

The objectives of the CCAFS regional scenarios development process are focused on policy engagement and planning and quantification of the scenarios by global models balances regional priorities and perspectives within a global context. Quantified scenarios provide a tool to measure and examine the relative impacts of regional socioeconomic development, for instance how population growth can affect ecosystems through the expansion of cropland. We use the stakeholder generated information from the trend indicators and narratives as the main link between the qualitative scenarios and the models. After establishing a quantitative link, we run the models over the time period and examine the impacts of the scenario assumptions. In the following paragraphs we describe several steps taken to interpret the scenario trend indicators from symbols (+ and −) into numerical values to be used as drivers in both models.

##### 2.3.1.1 Selection of indicators to quantify as drivers and ensure consistency of trends among scenarios

Of the full set of scenario indicators, we selected and quantified those which significantly impact the agriculture sector due to the importance of the sector in the region and the detailed representation of the sector within the models: population, GDP, technology-driven improvements in crop and livestock yields, and farm input costs. Because smaller groups of stakeholders directly gave the logic, direction, and magnitude of change over different time periods for each indicator, checks and adjustments were made to the trends the using the logic and scenario narratives to ensure that the trend indicators were consistent across the scenarios. The full set of indicators can be found in Table B1 in the Appendix in Supplementary materials.

##### 2.3.1.2 Compare scope of interest for both sets of indicators

As a next step, we mapped similar indicators of both the CCAFS and SSP scenarios, for example, “gross domestic product (per capita)” from the CCAFS indicators and “growth per capita and population growth” from the SSPs (O’Neill et al., 2015). In Table 1 (in Section 3) we present the mapping of the selection of the CCAFS scenarios indicators (column 1) with indicators for the SSPs (columns 9 and 11). While only a subset of indicators were selected to quantify and use as model drivers, we have mapped each CCAFS indicator to the SSP indicators in Appendix Table B2 in Supplementary materials.

**Table 1:** CCAFS scenarios trend indicators compared with and mapped to shared socioeconomic pathways and indicators.

Note: The first seven columns represent the stakeholder produced logic and direction of change for indicators of the CCAFS West Africa scenarios process that were used to develop the model drivers, with adjustments made to ensure consistency among the scenarios. The CCAFS indicators appearing in column 1 fall within same scope as the SSP indicators from columns 9 and 11. For a complete mapping of all CCAFS indicators and SSP indicators see Appendix Table B2 in Supplementary materials. An additional example of the mapping of indicators between scenarios can be found Appendix Table B3 in Supplementary materils.

##### 2.3.1.3 Mapping the CCAFS scenarios onto the SSP scenarios

There are some key differences between the regional West Africa scenarios and the SSPs. The SSPs were created by a community of researchers; the regional West African scenarios were created by a transdisciplinary group of regional stakeholders. However, for linking scenario sets, content matters most. Here, the main difference is that the SSPs have been framed in terms of their consequences for adaptation and mitigation, while the West Africa scenarios have been framed by their drivers − in this case, dominant modes of governance. The SSPs are defined by the level of challenge to climate adaptation and the level of challenge to climate mitigation and constructed upon two axes where the end points of each axis, high and low respectively, combine to define the “challenge space” of the scenario (O’Neill et al., 2014). This difference in framing makes the two sets of scenarios comparable rather than coherent in Zurek and Henrichs (2007) terms, but coherence between the socio-economic assumptions in the scenarios can be established − mapping the regional and global scenarios to each other through their narratives. We employed a “one-to-one” mapping system (Zurek and Henrichs, 2007) guided by the narratives and trend indicators to map the CCAFS scenarios onto the SSPs within the context of the SSP narratives (O’Neill et al., 2015). We present the results of the mapping of each CCAFS scenario to an SSP in Section 3.1.

##### 2.3.1.4 Quantifying the indicators in the context of SSP drivers

The quantification of the drivers of the SSPs that focus on the challenges associated with the socioeconomic development have been well-documented and provide insights into population and urbanization (Jiang and O’Neill, 2015, Kc and Lutz, 2014) and economic growth (Crespo Cuaresma, 2015, Dellink et al., 2015, Leimbach et al., 2015). Global integrated assessment models (IAMs) used the major drivers of the SSPs (such as population and income growth) to produce the first set of fully quantified global SSPs (Calvin et al., 2016, Fricko et al., 2016, Fujimori et al., 2016, Kriegler et al., 2016, O’Neill et al., 2015, O’Neill et al., 2014, van Vuuren et al., 2014). The modeling teams offered interpretations of the key elements of the narratives presented in O’Neill et al. (2015) as model inputs (crop and livestock yields; (Fricko et al., 2016, Herrero et al., 2014); energy sector (Bauer et al., 2016)) and also as model outputs (agricultural land use change (Popp et al., 2016); air pollution (Rao et al., 2016)). After review, we chose to use these SSPs drivers as a boundary condition or envelope of possible values (van Ruijven et al., 2014). Following the mapping of the CCAFS scenarios onto the SSPs, we used the value of the driver of the respective SSP as a starting point. Then, we used the trend indicators to guide and shift these values while making a critical comparison between both sets of narratives. The SSP indicator assumptions are defined for the end of the century rather than the CCAFS time period of 2050, this was taken into account when adjusting the trends. A detailed look into the scenario drivers is found in Section 3.2.

#### 2.3.2 GLOBIOM and IMPACT

GLOBIOM and IMPACT are global partial equilibrium models with a detailed representation of the agricultural sector. The similarities and differences between modeling approaches, of GLOBIOM and IMPACT in particular, have been examined through the intercomparison activities of the AgMIP project with focused reviews of the modeling of agricultural systems to meet future food demand (Valin et al., 2014), the impacts of climate change (Nelson et al., 2014b), and land use change (Schmitz et al., 2014). The quantification of the CCAFS scenarios benefits from the use of both models owing to their differences modeling approaches. Outputs from the scenarios modeled by GLOBIOM may prove useful as an input for modeling of regional impact assessments because the model considers multiple management systems, or *technologies*, the biophysical environment of production, or *climates*, and the *socioeconomic* context of the region (Antle et al., 2015, Havlík et al., 2015, Leclère et al., 2014). IMPACT has a long history of scenario analysis of alternative futures in the global agriculture system, and recent modeling improvements have expanded the commodities and countries that can be directly analyzed (Nelson et al., 2010, Robinson et al., 2015). Appendix Table C1 in Supplementary materials presents the main similarities and differences between both models used for quantifying the scenarios.

#### 2.3.3 Climate change impacts

West Africa is highly dependent on agriculture, predominantly rainfed agriculture, which makes the region particularly vulnerable to a changing climate. The strictly biophysical impacts on crop production due to changes in climate have been examined extensively within the model intercomparison projects, AgMIP and ISI-MIP, through globally-gridded crop models (Müller and Robertson, 2014). For West Africa, analyses of impacts, through crop models, as well as through empirical study, find that the negative impacts of climate change on crop yields are consistently negative across the climate and crop modeling results, though the magnitude of impacts remains uncertain (Jalloh et al., 2013, Müller et al., 2011, Müller and Robertson, 2014, Müller, 2011, Roudier et al., 2011, Sultan et al., 2013).

The biophysical effects from climate change on agriculture are applied here consistently with the SSP/RCP framework, which does not contain an explicit link between the socio-economic scenarios and climate change impacts, but rather suggests to test the different climate change scenarios under several socio-economic scenarios. van Vuuren et al. (2014), Rothman et al. (2014) and O’Neill et al. (2014) suggest that climate related biophysical factors should not be elements of the SSPs, but used in combination with SSPs and climate policies to define an integrated scenario. In the CCAFS scenarios, climate impacts are not “better” or “worse” among the scenarios, rather, climate impacts are examined as a force outside the scenario. We consider the projections of four General Circulation models (GCMs) available through the ISI-MIP project (Rosenzweig et al., 2014, Taylor et al., 2011, Warszawski et al., 2014) along with a constant 2000 climate. RCP 8.5 was selected because together with the current climate scenario it allows the scenarios to explore the most extreme trend envisaged for climate futures averaged across multiple crop models and assumptions of the impacts of CO_2_ response. RCP 8.5 is combined with SSP2 for the socioeconomic assumptions for the rest of the world. In principle RCPs and SSPs are completely independent dimensions (van Vuuren et al., 2014). However, more recent quantification of emissions under SSP2 in model ensemble suggest RCP 8.5 could be pessimistic under SSP2 economic development, although not impossible (Collins et al., 2013, Fricko et al., 2016, Raupach et al., 2007, Sheehan, 2008, van Vuuren and Riahi, 2008). It is worth noting that the range of climate effects for temperature increases of RCP 8.5 overlaps with the range for RCP 6.0 in 2050 (+0.8–1.8 for 6.0 and +1.4–2.6 for 8.5 in 2050) (IPCC, 2013, Riahi et al., 2016).

Using simulations of crop growth from two process-based globally-gridded crop models that consider the conditions of the future climates, we apply the relative differences of crop growth due to climate to the crop yields (Leclère et al., 2014, Mosnier et al., 2014, Müller and Robertson, 2014, Nelson et al., 2014b). The scientific community has yet to reach an agreement on whether the potential benefits from increases in CO_2_ can be taken up and used by crops, especially if temperature and precipitation reduce crop yields, therefore we consider a multi-model approach by including two globally gridded crop models with different assumptions on CO_2_ fertilization. The impacts of CO_2_ fertilization on crop yields is included in the EPIC (Environmental Policy Integrated Climate Model) crop modeling simulations used within GLOBIOM, while IMPACT simulates climate impacts without CO_2_ fertilization using DSSAT (Decision Support System for Agrotechnology Transfer). Taken together the yields from GLOBIOM and IMPACT can show the potential range of the biophysical and economic impacts on crop yields from climate change (more in Appendix F).

#### 2.3.4 Representative agricultural pathways (RAPs)

**Fig. 2:**
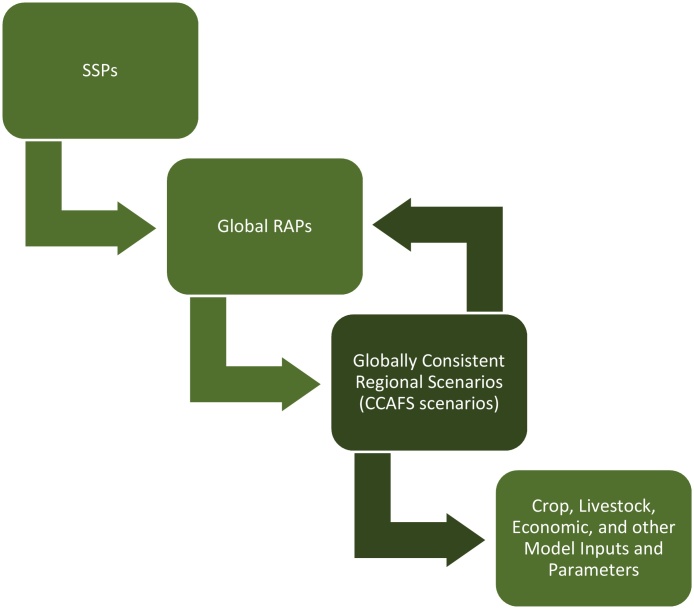
Globally consistent regional scenarios adapted from Valdivia et al. (2015).

National and subnational impact assessments that represent farm systems and households of small geographic units often require a globally consistent market equilibrium for commodities, which are produced by global or regional economic models (Valdivia et al., 2015). The integrated scenarios of the SSPs/RCPs provide modeling communities with the global, and to some extent the regional, sector-specific storylines. These storylines as they pertain to agriculture serve as global representative agricultural pathways (RAPs). At the same time, researchers focusing on adaptation challenges have devised some local RAPs specific to particular contexts in Africa, although these are disconnected from a global consistent framing (Antle et al., 2015, Valdivia et al., 2015). The CCAFS scenarios, quantified by GLOBIOM and IMPACT, examine regional stakeholder development pathways within the space of the SSPs offering the first globally coherent, *regionally relevant* RAPs. The perspective and examination of the possible development of the region through the lens of regional stakeholders can provide feedback to the global RAPs as well as consistency for downscaled scenarios (Fig. 2).

## 3 Results

First we present the mapping of the CCAFS scenarios to the SSP scenarios (Section 3.1) and the quantified drivers of change for the CCAFS scenarios (Section 3.2). We then highlight the impacts of the scenarios on improving food security, the regional supply of crop and livestock products and impacts on the environment including land use change (Section 3.3). Though the regional scenarios were defined by uncertainty concerning the most active actors and long-term versus short-term priorities, we make an assessment of the vulnerability of the scenarios, completing the link of the regional scenarios to the SSPs which are defined by the challenges to adaptation (Section 3.4).

### 3.1 CCAFS scenarios in the context of the SSPs

In *Self-Determination*, where strong state actors focus on long-term issues, trend indicators align closely with *SSP1: Sustainability* in nearly all qualitative elements describing the SSP narrative, such as investments in productivity and extension services, increased education and health and sanitation services, regulations to reduce deforestation, and effective social protection schemes. A key difference is that investments are estimated to be lower in the CCAFS scenario due to a lack of financial support from outside the region and a reliance on regional resources. Additionally, within the CCAFS scenario the struggle for institutional change may open up the opportunity for corruption, which is inconsistent with *SSP1* where strong institutions are effective at the national and international levels. In figures, *Self-Determination* will appear as “SelfDet”.

In *Save Yourself*, action is not taken by the weak and unstable governments, but by CSOs in an emergency response manner, and by the private sector acting with short-term profitability interests, which mirrors the global narrative of *SSP3: Regional Rivalry*, of weak institutions, low technology development for the agriculture sector and food security issues due to growing inequality and high population growth. The CCAFS scenario reflects some aspects of the low-income country narrative of *SSP4: Inequality,* though the key difference is that the West Africa scenario sees more political instability and ineffectiveness of institutions which hinders the region’s development and access to markets while, *SSP4* represents a world where growing inequality (within and between countries) stems from limited access to education and consolidation of political and economic power by elites. Therefore we align *Save Yourself* with *SSP3*, and the scenario in figures, appears as “SaveYourself”.

*Civil Society to the Rescue*?, where weak governments are replaced with strong CSOs tackling food security with a long-term focus, together with strategic investments by a more socially conscious private sector, is most closely represented by *SSP2: Middle of the Road*, where some actions for protection lead to a decline in deforestation rates, modest productivity and commercialization benefits fall to those who already have capacity rather than inducing a transformation of smallholders, and moderate increases in education and health issues are largely taken up by CSOs with private sector support. Ultimately, in this scenario, the lack of government support and coordination means that non-state ambitions are only partially achieved. In figures, *Civil Society to the Rescue?* will appear as “CivilSociety”.

The short-sighted prioritization of governments interested in maintaining power in the *Cash, Control, Calories* scenario, creates a highly urbanized, high economic growth focused scenario, leading to reactive investments in education and health services, similar to *the SSP5: Fossil-fueled Development*. The difference with *SSP5* is that in this scenario, investment cycles are short, creating unstable development throughout the scenario period. Additionally, O’Neill et al. (2015) discussed the possibility that actions taken within a development pathway may change a pathway and alter the challenges for adaptation or mitigation, which can be seen in the “start and sputter” nature of *Cash, Control, Calories*, making the scenario quite different than *SSP5* by the end of the time period. In figures, *Cash, Control, Calories* will appear as “CCC”.

**Fig. 3:**
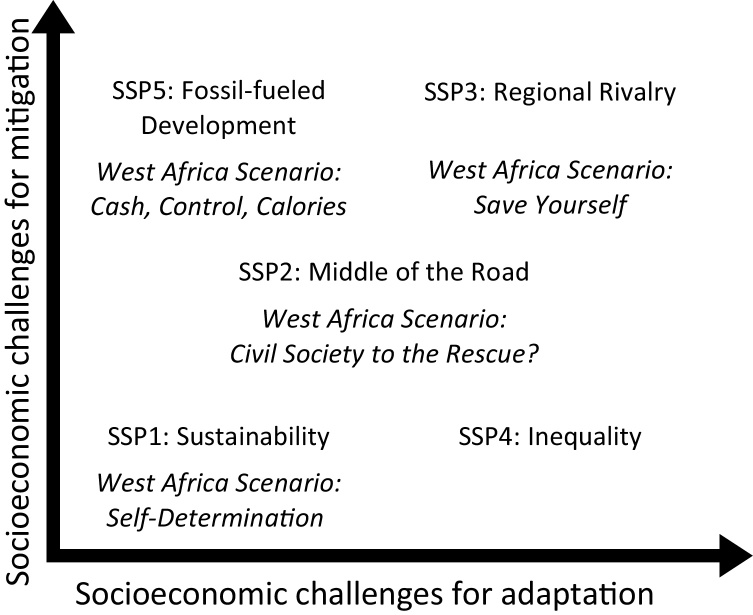
The five shared socioeconomic pathways (SSPs) mapped to the CCAFS West Africa scenarios.

Source: Figure adapted from O’Neill et al. (2015).

In our mapping of CCAFS scenarios onto the SSPs, we have looked for overlapping in the narratives and the storylines at the regional level, because we assume the rest of the world follows the *SSP2* storyline for all scenarios. Although the SSPs themselves are global in nature and scope, this allows us to examine the impacts of the regional assumptions. Fig. 3 illustrates the linkages between the stakeholder-defined scenarios for West Africa and the narratives of the SSPs from O’Neill et al. (2015) with the CCAFS scenarios appearing in italics.

### 3.2 Quantified CCAFS scenario drivers

In this section, we present the results from the methodology presented in Section 2.3.1 analyzing the following drivers: socioeconomic development, crop and livestock productivity, regional integration, expansion of cropland area, and the development of the rest of the world. Table 1 presents an overview of the four selected trend indicators that were translated from stakeholder information into numerical values to use within the models as drivers. Each indicator is grouped into four rows by scenarios with the direction and magnitude of change and logic for change as provided by the stakeholders. Columns 10 and 12 of Table 1, expand on the “one-to-one” scenario mapping presented in Section 3.1, offering insights into how well the SSP-specific indicator assumptions match with its corresponding CCAFS scenario, where green indicates a “good match,” yellow indicates a “neutral match”, and red indicates a “bad match.” When the indicator assumptions were “bad match” between SSP assumptions and the CCAFS indicator trends, we adjusted the SSP drivers to better match the CCAFS narrative storylines.

#### 3.2.1 Macroeconomic and socioeconomic development

Understanding regional economic development, population growth, the role of regional integration on agricultural inputs, and development outside of the region are essential to assess West Africa’s future development.

We compared the economic and demographic developments, a critical factor in determining food demand (Valin et al., 2014), for the region up to 2050 for the SSPs (Dellink et al., 2015, Kc and Lutz, 2014, O’Neill et al., 2015). Then, guided by the regional scenario narratives and the trend indications of change developed by the stakeholders during the scenario development workshops (first four rows of Table 1), we adjusted these drivers for the region to capture the uncertainty around governance and political stability inherent in the regional scenarios as they pose a challenge for development in Western Africa (Palazzo et al., 2016, Palazzo et al., 2014). In West Africa, the population of the region grows from 300 million in 2010 to almost 600 and 800 million in *Self-Determination* and *Save Yourself* respectively (Appendix D2 in Supplementary materials). GDP per capita increases across all scenarios, but by 2050 all scenarios remain lower than the regional SSP projections (Appendix D1 in Supplementary materials). *Cash, Control, Calories* initially sees the largest increase, but its GDP development is unstable, and it begins to slow and declines slightly after 2040–reflecting the short-termism of the scenario. Per capita GDP is the highest in *Self-Determination* by 2050 and *Civil Society to the Rescue?* experiences a steady and consistent increase in per capita GDP. Per capita GDP in *Save Yourself* increases the least amongst the scenarios over the time period and follows cycles of growth and recession, representing unstable economic development.

The impacts of the scenarios assumptions within the region are isolated to some extent by assuming that the global context in each of the scenarios follows the same trends for climate impacts, agricultural development, and socioeconomic development. In principle, underlying variables of demographic and economic development are correlated across regions (fertility rate, mortality, investment, technological adoption), however, some regional deviations are plausible, as political context also strongly influences the evolution of such variables. For easier comparability of the direct impacts of the regional drivers, we intentionally varied only the West Africa parameters, changing other parameters over time but keeping them constant across the scenarios for other regions of the world, similar to what was done in the CCAFS Southeast Asian Scenarios (Mason-D’Croz et al., 2016). The rest of the world follows the *SSP2* population and economic development trajectory where, by 2050, the global population reaches 9.2 billion people (Kc and Lutz, 2014) and global average GDP per capita doubles to around 16,000 USD (Dellink et al., 2015).

The degree to which current regional integration efforts within Africa and the ECOWAS community are struggling to find success in agriculture highlights the challenges facing the region (UNCTAD, 2012, United Nations, 2009). The CCAFS scenario narratives consider the challenges to regional integration, including the lack of regulation, which have been brought into the quantitative modeling of GLOBIOM through impacts in the farm input costs (rows 4–8 in Table 1 and Appendix D in Supplementary materials). Limitations in the trade of both the inputs to and products of agriculture and shocks in the agricultural supply chain, stemming from conflicts or climate change can have profound effects on food security (Baldos and Hertel, 2015, Mosnier et al., 2014, Simson and Tang, 2013, van Dijk, 2011). Conflicts are highlighted in each of the scenarios, although in *Save Yourself* the lack of strong state governments combined with short-term priority setting gives this scenario the most potential for food insecurity.

#### 3.2.2 Crop and livestock productivity

**Fig. 4:**
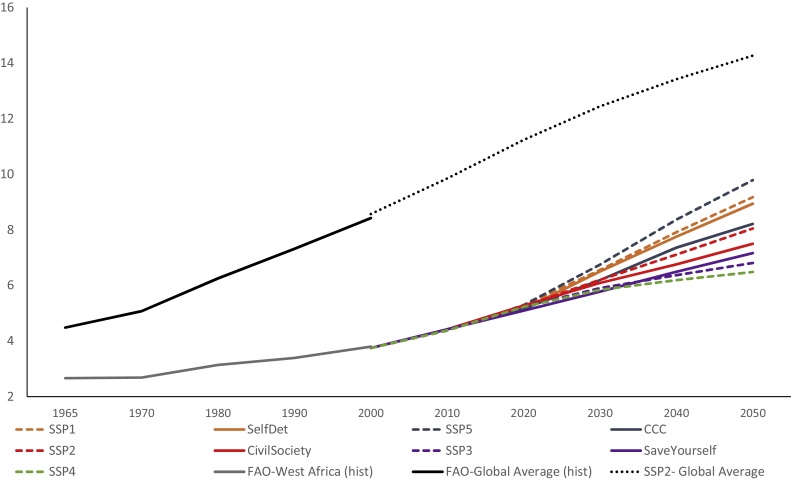
Historical and aggregate exogenous crop yields (gigacalories per ha) for CCAFS West Africa by scenario and for each SSP and historical and exogenous SSP2 global average.

Source: FAOSTAT (2016) for historical; Fricko et al. (2016) for the SSP2 global average; Authors for projections for West Africa scenarios.

Technical progress in crop production is represented in both models through increases in crop yields. To estimate crop productivity over the time, we use an econometric estimate of the relationship between crop yields and GDP per capita assumptions of the SSPs (Fricko et al., 2016, Herrero et al., 2014). The IAMs used in the quantification of the integrated SSPs project changes in crop yields, with different IAMs providing the “marker” for each SSP. For consistency, we have used the GLOBIOM yield projections for each SSP (as a starting point), and then made adjustments based on the scenario narratives and trend indicators (for agricultural productivity and crop-specific productivity). These trends appear in the last four rows of Table 1. In IMPACT, the crop yield trends were quantified by applying the scenario deviations from the GLOBIOM *SSP2* baseline to the IMPACT *SSP2* baseline, which were estimated based on historical yield trends, agricultural research and development, and assumptions on how these could change over time (Sulser et al., 2015). The gap between the global average yields and yields in West Africa will remain a challenge for the agricultural system even in the scenario with the highest investment in agriculture, *Self-Determination* (Fig. 4).

The contribution of the livestock sector to the national GDP ranges from 10% to 15% (Kamuanga et al., 2008). Recent livestock foresight studies spotlight the region’s potential in transitioning from extensive land based systems to mixed crop-livestock systems (Herrero et al., 2014), intensifying pastoral systems while also protecting pastoralists and animal health, echoing assessments made by the Sahel West African Club Secretariat and OCED (Kamuanga et al., 2008).

Productivity of livestock can be measured by the conversion efficiency of the quantity of livestock product produced per unit of feed consumed. We used the projections of conversion efficiencies for livestock for the SSPs (Herrero et al., 2014) as a starting point and further developed the projections using the narratives and indicator logics and trends (rows 9–12 of Table 1). The investments in ruminant production, due to the growing food demand as outlined in the scenario narratives, result in yield improvements in *Self-Determination*, while the focus on dairy production and monogastric production in the early decades of *Cash, Control, Calories* is considered. In *Civil Society to the Rescue?* meat demand drives the investments from private sector and social entrepreneurs. Little investment is made for livestock or veterinary services in the *Save Yourself* scenario resulting in relatively insignificant yield improvements.

#### 3.2.3 Cropland area expansion

To harmonize the quantitative modeling results, cropland area expansion as modeled by GLOBIOM was used as an input into IMPACT, although the distribution of crop area by crop type and management system, in this case irrigated or rainfed cropland area, remained endogenous (Appendix D in Supplementary materials). Cropland in the region expands nearly 55% in *SSP2* by 2050. In *Cash, Control, Calories* and *Self-Determination* cropland increases less (51% and 46%, respectively) and *Save Yourself* and *Civil Society to the Rescue?* cropland increases slightly more (59% and 57%, respectively).

### 3.3 Quantified CCAFS scenarios

By applying the changes in the scenario drivers for the region over time within the models we provide a plausible future of the regional development of the agriculture sector, both in the demand and supply of products as well as the competition for land for agricultural production. In the following sections, we summarize the scenario results as they pertain to crop and livestock production (Section 3.3.1), food availability, prices, and net trade (Section 3.3.2), and land use change (Section 3.3.3). While this paper focuses on the multiple, plausible futures of socioeconomic development of West Africa, the development of the rest of the world follows the trends of *SSP2* (Fricko et al., 2016). Economic growth improves food security spurring an increase in the production of crop and livestock products globally. We have highlighted some of the ways the development of the region affect the rest of the world in Appendix H in Supplementary materials. The changes in cropland area expansion from GLOBIOM were used as an exogenous driver in IMPACT, therefore the scenario results presented in this section that explore the expansion of cropland area regional land use change were modeled by GLOBIOM.

#### 3.3.1 Agricultural production and climate impacts

**Fig. 5:**
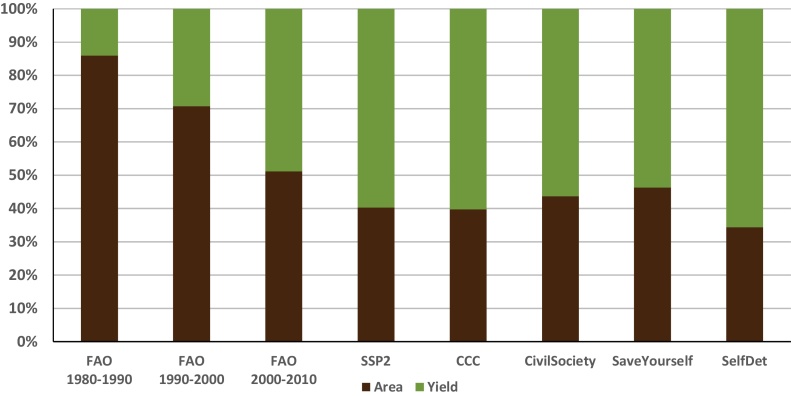
Share of the source of production growth based on the rate of growth for West Africa over historical trends and scenario projections for the CCAFS scenarios and SSP2. Note: Area is cropland area expansion and yield is the increase in the aggregate crop yield in tons per hectare.

Source: FAOSTAT (2015), (left side); GLOBIOM model results (average over 2010–2050) (right side).

Agricultural production currently accounts for about a quarter of the region’s GDP, but was as high as 35% in the 1980s (World Bank Development Indicators, 2015). West Africa, as a region, is the leader, or among the top global producers of cassava, millet, sorghum, and oil palm (FAOSTAT, 2015). Historically, increases in production within the region have come from expanding cropland area rather than through significant yield improvements (Byerlee et al., 2014, Fischer et al., 2014, Hillocks, 2002). In the CCAFS scenarios, the historical trend continues in the *Save Yourself* and *Civil Society to the Rescue?* scenarios from 2010 to 2050, where slightly less than half of the average annual growth in production comes from crop area expansion. Alternatively, almost 66% of the increase in production in the *Self-Determination* scenario comes from yield improvements (Fig. 5).

In both models, crop production in the region increases from 2010 to 2050 for all scenarios, with *Self-Determination* having the highest levels of crop production and *Save Yourself* having the least growth in crop production (Appendix Fig. E2 in Supplementary materials). The development of crops in the region remains of particular importance to the global production by 2050, especially for millet, cassava, and sorghum (more details in Appendix E in Supplementary materials).

Investments in livestock production nearly quadruples the total livestock calories produced (from dairy, ruminant and monogastric meat) for *Cash, Control, Calories* and *Self-Determination* in GLOBIOM and triples in IMPACT. Although there is little investment in the livestock sector (aside from the dairy sector) in *Save Yourself* and limited investment in *Civil Society to the Rescue*?, these scenarios still see an annual increase of total livestock production of between 2.3% and 2.8% per year, respectively in both models.

**Fig. 6:**
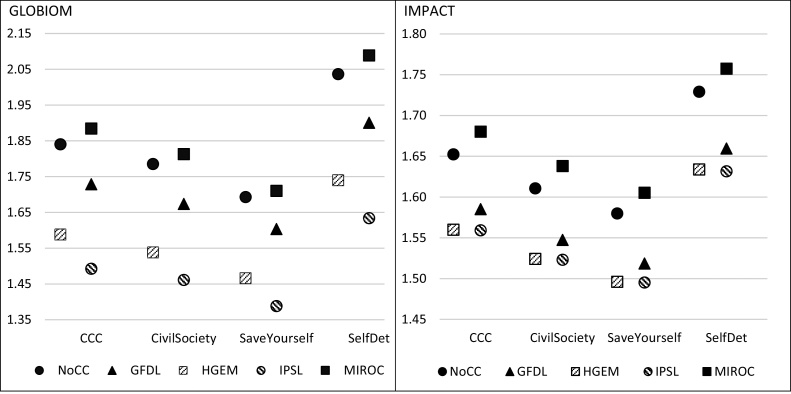
Relative change in average crop yields in 2050 compared to 2010 yields as modeled by GLOBIOM and IMPACT for the CCAFS West Africa Scenarios with and without the climate change effects on crop growth included. Note: The y-axis is not the same for both models.

Source: GLOBIOM model results (left side); IMPACT model results (right side).

Examining the impacts on the most important crops to the region shows that, on average, climate change lowers crop yields (Fig. 6). This is consistent with other assessments under varied climatic conditions using West Africa specific crop models, despite one of the GCM climate models (MIROC) predicting conditions where climate change is more favorable to crops (Sultan et al., 2013). Aggregated crop yields provide a rough estimate of the impacts of climate change, however, these may underestimate the impacts to individual crops (such as millet, sorghum, and cassava). The impacts for individual crops can be found in Appendix Table F2 in Supplementary materials. For both models, the negative climate impacts on aggregate crop yields in the *Self-Determination* scenario, which has the highest exogenous yield improvements, are in most cases, still greater than the yields for the three other scenarios without climate impacts, suggesting that adaptation measures and investments taken in the present can lessen the impacts of future climate change. The flexibility of the endogenous area reallocation response within GLOBIOM makes the model more responsive to the yield effects of climate change than IMPACT (Nelson et al., 2014a, Nelson et al., 2014b).

#### 3.3.2 Markets and food demand

**Fig. 7:**
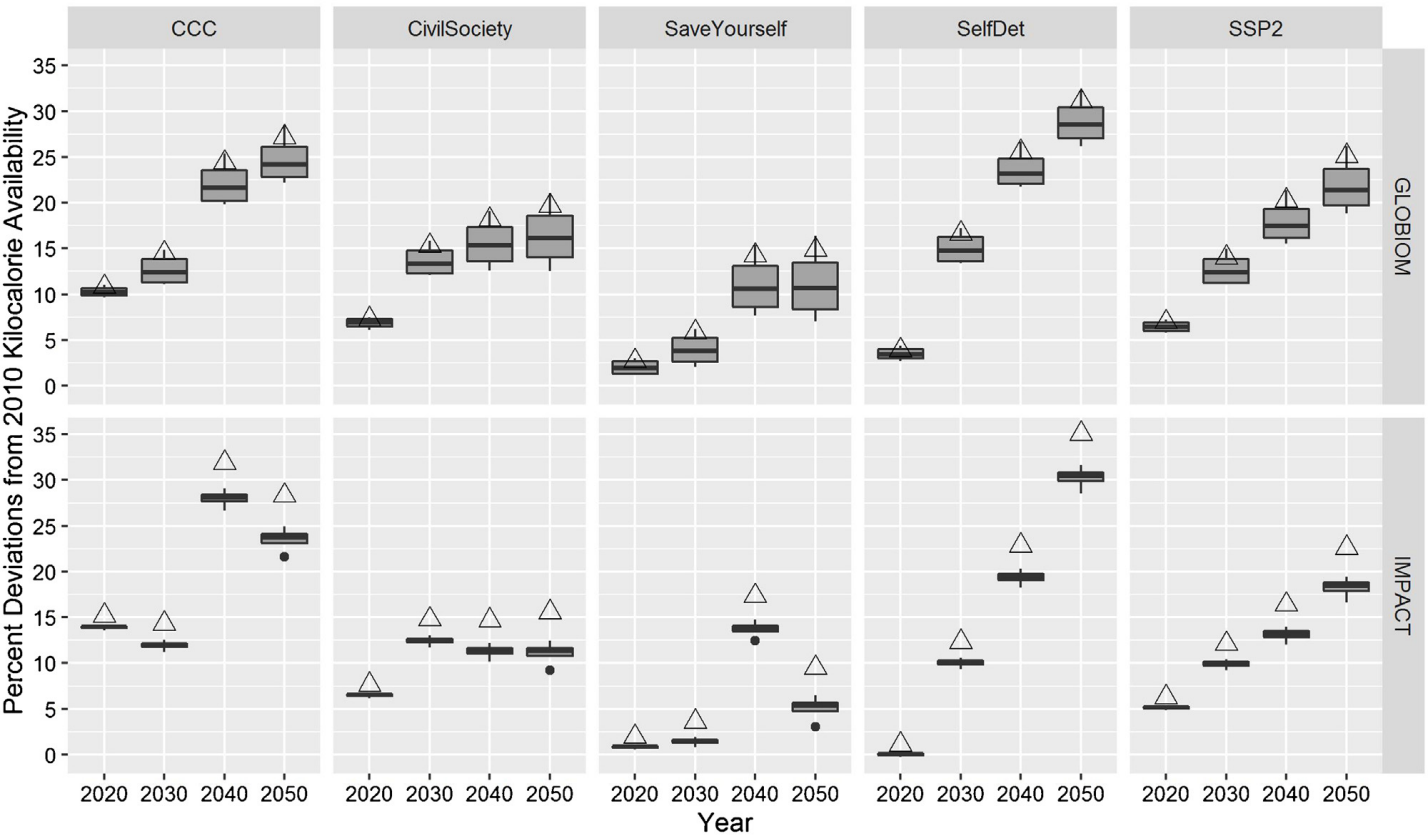
Percent deviation in kilocalorie availability per capita per day from 2010 values for the CCAFS West Africa scenarios and SSP2 under no climate change (triangles) and under the effects from climate change (box plots). Note: The box plots represent the spread of calorie availability for each scenario under climate change.

Source: GLOBIOM model results (top row) and IMPACT model results (bottom row).

Kilocalorie availability per capita per day, a commonly used indicator to measure food security, considers the total food products demanded by a region and translates the quantity of product to calories. As the income per capita increases over the time period in all scenarios, food demand, and kilocalories available, increase in the region (Fig. 7). *Self-Determination* sees the greatest improvement in food security due to the long-term prospective and high economic growth. *Cash, Control, Calories*, with a relatively large increase in the GDP per capita, sees a limited improvement in food security due to the nature of markets within the region. Food security remains a challenge for the region in *Save Yourself* due to the relatively low economic growth and high population growth and failing state of the region’s agriculture. The SSP that maps to *Save Yourself*, *SSP3*, was also found to present challenges for the food security in Africa in other quantitative assessments (Hasegawa et al., 2015). In terms of the diet composition, the scenarios with the highest economic growth and largest investment in livestock productivity*, Cash, Control, Calories* and *Self-Determination,* have the largest consumption of meat products. *Civil Society to the Rescue?* and *Save Yourself* have a smaller decrease in the per capita demand for cassava and other tubers than in the other scenarios, which is consistent as cassava is a staple food crop typically consumed less with rising incomes, and is already seen in cities in the region (Appendix Fig. G3 in Supplemenatry materials).

By 2050, the average price for crops increases over time for *Save Yourself* and *Civil Society to Rescue?* while decreasing for *Cash, Control, Calories* and *Self-Determination*. However, climate change increases the average prices for all crops (for 3 of the 4 GCMs). In 2050, prices under climate change increase additional 15%, on average, in *Save Yourself*, though only 4% in *Self-Determination*. Appendix F in Supplementary materials examines in more detail the variability of prices from GLOBIOM in the scenarios and under climate change.

GLOBIOM and IMPACT model results agree that net imports of crops, as a share of the regional production, are highest for *Save Yourself* and *Cash, Control, Calories* and lowest for *Self-Determination*. The model results agree also that imports of all livestock products increase in the region over time, however there is no agreement in the scenario that will have the largest imports as a share of the regional production.

#### 3.3.3 Agricultural area expansion and land use change

Increases in food demand are met either through productivity increases or though expansion of crop and grassland. Demand not met by regional production will be met by increased production from outside the region. Shifting agricultural expansion outside the region has possible unintended environmental effects. Any agricultural expansion can affect land use within West Africa, outside West Africa but within Sub-Saharan Africa, and in the rest of the world.

**Fig. 8:**
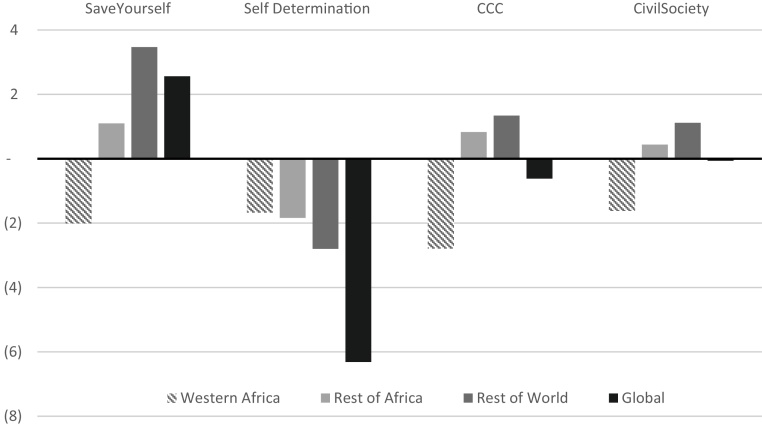
Difference in forest and natural land converted to agricultural land (cropland and grassland) from 2010 to 2050 as compared to SSP2 (M ha) Note: Negative values imply land sparing compared to SSP2.

Source: GLOBIOM model results

Globally, agricultural area (cropland and grassland) expands more than 11% in *SSP2* by 2050 (Fig. 8). The Green Revolution, where the adoption of improved seeds increased agriculture output worldwide, is credited with saving, over forty years, at least twice as much land from being converted in developing countries (Stevenson et al., 2013). While relatively small, the differences in agricultural area between *SSP2* and the regional scenarios, reflect the impacts of the region’s thriving or struggling development on the rest of the world: comparative savings of 6.2 million ha (*Self-Determination*) or an additional conversion of 2.6 million ha globally (*Save Yourself*).

Within West Africa, land converted for use by agriculture is higher in *Self-Determination* than in the other scenarios suggesting that the market conditions and productivity gains increase the sector’s profitability and may incentivize expanding cropland and grassland, in what is known as Jevon’s paradox (Alcott, 2005, Byerlee et al., 2014, Hertel et al., 2014). However, the thriving agriculture sector of the *Self-Determination* saves almost 3.64 ha outside the region for every 1 ha converted within the region, and, on average, half of the unconverted agricultural area is saved from within other African regions. In *Save Yourself*, the region’s agriculture sector struggles, farm input prices increase and less land is used for agriculture in the region by 2050, but at the expense of additional agricultural area converted globally. In the integrated assessments of the SSPs, *SSP3*, the match of *Save Yourself*, was also found to put the most pressure on land resources whereas *SSP1*, the match to *Self-Determination,* reduced the global burden on land (Popp et al., 2016, Riahi et al., 2016).

### 3.4 Challenges to adaptation

O’Neill et al., 2014, O’Neill et al., 2015 define the SSPs by the combination of the socioeconomic challenges to adaptation and to mitigation, and assert that the elements that describe these challenges to adaptation are growth in income, effectiveness and access to institutions, infrastructure and barriers to trade, and human capital.

Rothman et al. (2014) implore that the challenges to adaptation take an integrated perspective and consider outcome and contextual perspectives. As such, we consider indicators that, taken together, can assess West Africa’s vulnerability to climate change. Adger et al. (2003) define the vulnerability of a system to climate change by “its exposure, by its physical setting and sensitivity, and by its ability and opportunity to adapt to change.” (p.181) We posit that the collective examination of the sensitivity of the indicators to change, over the time period − considering the underlying scenario assumptions combined with or absent from the biophysical impacts of climate change − implies that a given scenario will lead to higher or lower vulnerability and thus face higher or lower challenges to adaptation, relative to the other regional scenarios. The ranking of scenarios among the indicators, from low vulnerability to high vulnerability, is presented in Appendix I in Supplementary materials.

**Fig. 9:**
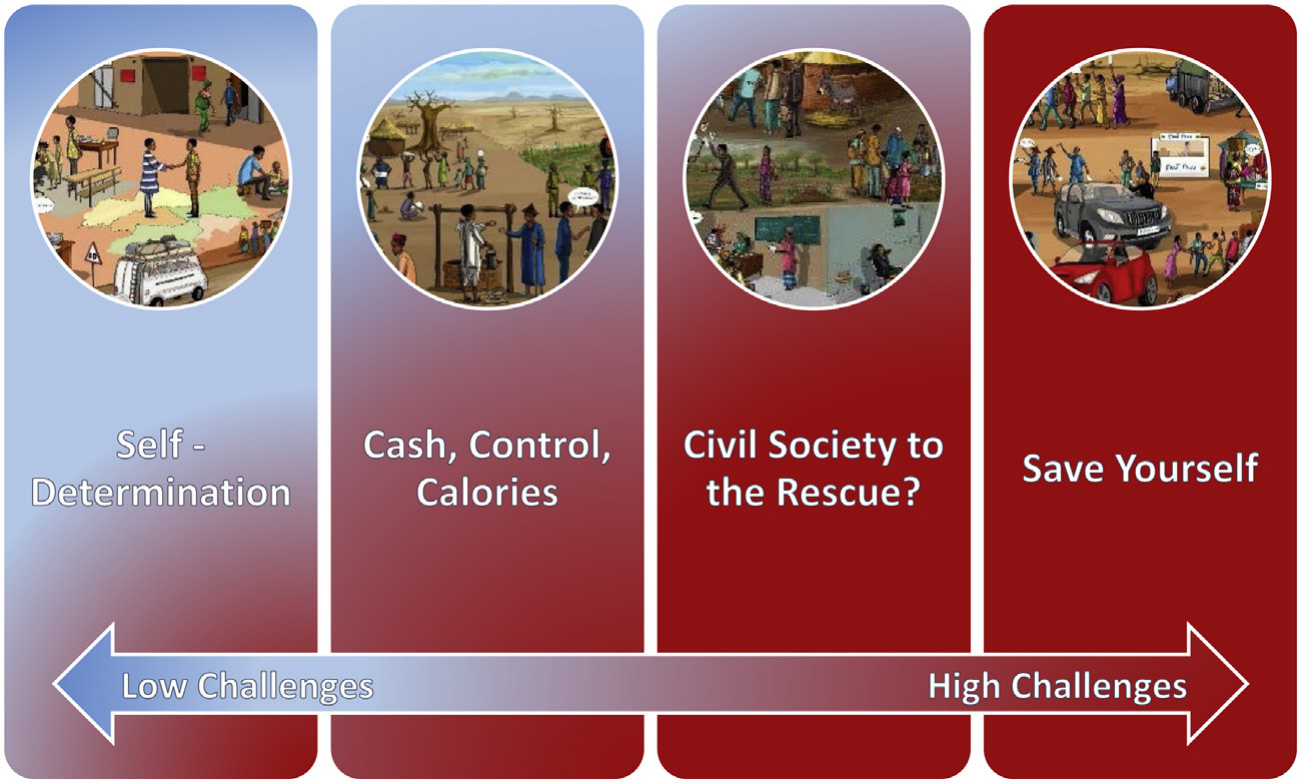
The CCAFS West Africa scenarios presented along the challenges for adaptation axis. Note: Scenarios presented toward the left (right) side of the challenge space represent scenarios with lower (higher) challenges to adaptation.

Source: Authors; figure adapted from O’Neill et al. (2015); Drawings by artist Andre Daniel Tapsoba

In Fig. 9, we adapt and expand on the assessment of the SSPs along the axis of challenges to adaptation found in Fig. 3(b) from O’Neill et al. (2015) to include the CCAFS West Africa scenarios along the axis. We find that most of the regional scenarios, when ranked by vulnerability, fall within their respective SSP challenge space for adaptation; *Self-Determination* faces the lowest challenges to adaptation and *Save Yourself* the highest challenges to adaptation. For the other two scenarios, when ranked, they fall in the range of intermediate challenges to adaptation. In *SSP5: Conventional Development*, the partner of *Cash, Control, Calories*, it is assumed that the challenges to adaptation are low and that only the mitigation challenges dominate, but the nature of the regional scenario (“start-and-stop”) creates challenges for longer-term adaptation. In *Civil Society to the Rescue?* adaptation challenges are primarily related to a lack of key capabilities among regional actors because of a lack of active government support.

## 4 Discussion and conclusions

### 4.1 Choices in linking regional scenarios and SSPs

The focus of the SSPs has been on global pathways and dynamics offering limited insights at national and regional levels. The quantified socioeconomic storylines of the SSPs have been provided for use at the national and sub-national levels, but insights into the impacts and drivers of plausible future regional developments have been lacking − and this is especially the case for the West African region. Rather than exploring impacts on the region of the multiple stories of global development, as the integrated SSPs provide, the regional scenarios examine multiple stories of regional development, that are linked to the regional assumptions of the SSPs, within a single global pathway, in this case *SSP2*.

The region is a key level at which to develop scenarios − because it allows for a connection to the global level and scenario assessment while still being relevant to regional economic bodies like ECOWAS, as well as to national governments (Biggs et al., 2007, Zurek and Henrichs, 2007). Because the scenarios offer multiple, challenging contexts in which to test draft plans and policies, they have been used by the private and public sector in Burkina Faso to review the country’s National Plan for the Rural Sector (PNSR) and in Ghana to guide and inform district and national level policy processes including a review of the National Livestock Policy. Plausible futures of the development of the agricultural system at the regional level can also provide appropriate and necessary inputs for more disaggregated impact assessment (Antle et al., 2015, Valdivia et al., 2015). Additionally, linking the scenarios between levels allows policy makers to address issues within their decision contexts. Appendix J in Supplementary materials provides additional details on the use of the regional scenarios for West Africa presented in this paper that have led to policy change.

We have presented an example in which regional scenarios have been created and linked to the global SSPs. We use the Zurek and Henrichs (2007) framework for linking scenarios across levels to characterize the connection between the West Africa scenarios and the global SSPs starting as ‘comparable’ – meaning that the subject matter between the two scenario sets is similar in scope, and, through the quantification process, moving toward ‘coherent’ – meaning that the two sets of scenarios can be mapped to each other in terms of basic assumptions. It is useful to consider this choice in the context of other possible choices. ‘Equivalent’ scenarios at SSP and regional levels are not often a desirable option – since this means ‘downscaling’ the global SSPs without any consideration for local context, though exceptions are possible, for instance when the goal is to build a direct local version of a global scenario set (Kok et al. In Review). ‘Consistent’ scenarios, where the main assumptions of the SSPs are directly downscaled to the regional level, but where some elements of the scenario context may be changed because of regional contextualization, is a viable option, as per Jalloh et al. (2013), offering clear links between regional and global scenarios and strong comparability across regions. Downscaling is also less time intensive than creating a new set of scenarios – regional stakeholders only need to interpret what each scenario means at their level, rather than having to conduct their own driver analysis and create their own scenario framework. However, there is no guarantee that such a dominant SSP-based framing responds usefully to regional policy priorities if they are not defined by regional policy makers. Since the presented set of scenarios was primarily targeted at regional policy guidance, we chose to start with an independently framed, ‘comparable’ set of scenarios, focusing on similar issues as the SSPs, but with different basic assumptions. By mapping the regional scenarios to the SSPs through quantification, ‘coherent’ elements were added, allowing for a global contextualization of a primarily regionally focused scenario set. If we had created a completely ‘independent’ scenario set, with no similarity in focus to the global SSPs and no process to create coherence between levels, it would not have been possible to provide a suitable global context to the scenarios.

Furthermore, integration of the quantitative results back into the basic qualitative scenario narratives was only done whenever the qualitative scenarios were used for policy guidance, and quantitative results were presented and checked for coherence with the narratives. Integration of insights from scenario quantification into the basic regional scenario narratives would have made them more fully coherent from the start. This would be especially recommended if scenario quantification is used to investigate the impacts of different global scenarios on regional conditions.

### 4.2 West African agricultural, food security and climate futures

The scenarios also offer the opportunity to reflect on the potential agricultural, food security, and climate futures of the ECOWAS region as well as its socioeconomic developments. In the future, food security may pose a challenge when population grows rapidly and is coupled with stagnate or unstable economic growth. Long term priority setting that focuses on economic growth increases food availability − however, the quantitative models are not yet equipped to model income inequality or urban and rural poverty.

Climate change is likely to have a negative effect on both crop yields and grassland productivity, and the lack of investment in crop productivity may exacerbate the challenges of climate change. Crop prices suffer from greater shocks in general under futures with little investment in crop productivity, in particular millet and sorghum. This calls for action now to both implement incremental adaption that will improve the resilience of populations, but also to plan transformational adaptation timeframes which will outline when appropriate research and policy changes need to be put in place in order to maintain production levels and avoid placing food security and smallholder farmer livelihoods in jeopardy (Rippke et al., 2016).

The scenario development process laid out here produced a wealth of qualitative information from stakeholders, some of which fell outside the scope of the models used to quantify and simulate them. For example, indicators on changes in inequality, and the access to and quality of health and human services. Other topics discussed (i.e. land use, prices, etc.) could not be applied directly because they were endogenously resolved in the models. Nevertheless, the discussion revolving around these many topics was still useful, and contributed to the rich scenario narratives, which may yet serve as starting point for future work. This could include expanding the modeling suite to include new tools to expand the current analysis to challenging questions such as the dynamics behind urban and rural poverty, and inequality with respect to access to food.
