# Supplementary material for: Linking regional stakeholder scenarios and shared socioeconomic pathways: Quantified West African food and climate futures in a global context
Source: Glob Environ Change. 2017 Jul;45:227–42. doi: 10.1016/j.gloenvcha.2016.12.002 (PMC5637935; doi:10.1016/j.gloenvcha.2016.12.002)
Supplement: Supplementary file 1 [file mmc1.pdf]

Appendix Contents

A. Development of the CCAFS scenarios ... 3
Scenario development ... 3
Participants of the CCAFS scenario development workshops ... 3
Figure A1. Short narrative descriptions of the scenarios along the axes of uncertainty: a) will short-term priorities or long-term priorities be the focus of governance and b) will state actors or non-state actors be the driving force in the region? ... 4
B. Comparison of the CCAFS Regional Indicators with the SSP Indicators ... 5
Table B1. Trend indicators of interest for the CCAFS scenarios ... 5
Table B2. Mapping of CCAFS semi-quantitative indicators and SSP indicators. ... 6
Table B3. Comparison of a selection of the CCAFS indicators with the SSP indicators using the qualitative information, logic, and trends ... 8
C. Model Comparison ... 9
Table C1. GLOBIOM and IMPACT comparison ... 9
D. Model Drivers ... 10
Cropland area expansion ... 10
Regional Integration Impact on Farm Input Costs ... 10
Socioeconomic drivers ... 10
Figure D1: Relative change in GDP per capita for West Africa 2000-2050 for the CCAFS scenarios and SSPs (2000=1) GDP values from OECD (Dellink et al., 2015) and population values from IIASA (Kc and Lutz, 2014) ... 11
Figure D2: Population of Western Africa in millions of people for the CCAFS scenarios and SSPs (population values from IIASA (Kc and Lutz, 2014)) ... 11
E. Crop and livestock production and share of global production ... 12
Figure E1. Western Africa's share of crop production by crop (large circles) and the region's production share of global production by crop (smaller circles) in dry matter for 2010 (top most figure) and in 2050 by scenario (figures in lower four rectangles) Source: FAOSTAT 2015 (2010); GLOBIOM model results (2050). ... 15
Figure E2. Growth in production of selected crops in Western Africa by CCAFS scenario and SSP2 from 2010 to 2050 in million dry matter tons from GLOBIOM ... 16
Figure E3. Millet average annual growth in production from FAO statistics (FAOSTAT, 2015) and CCAFS scenarios from 2010-2050 the growth in production is allocated to either expansion of area or improvement in yields ... 16
Figure E4. Sorghum average annual growth in production from FAO statistics (FAOSTAT, 2015) and CCAFS scenarios from 2010-2050 the growth in production is allocated to either expansion of area or improvement in yields ... 17
Figure E5. Maize average annual growth in production from FAO statistics (FAOSTAT, 2015) and CCAFS scenarios from 2010-2050 the growth in production is allocated to either expansion of area or improvement in yields ... 17

Figure E6. Cassava average annual growth in production from FAO statistics (FAOSTAT, 2015) and CCAFS scenarios from 2010-2050 the growth in production is allocated to either expansion of area or improvement in yields 18

F. Applied Climate Change impacts in GLOBIOM and IMPACT 19

Table F1 Climate Scenarios 19

Table F2. Crop yields indexed to the 2010 values with the endogenous scenario impacts and climate effects for a selection of six crops by model 21

G. Per capita demand, prices, and net trade 25

Variability in GLOBIOM Prices by Scenario (over time and under climate change) 25

Figure G1a. Deviations from the 2010 indexed price for crops for all GCM simulations and decades from GLOBIOM 27

Figure G1b. Deviations from the 2010 indexed price for crops for all GCM simulations and decades from GLOBIOM (without millet and groundnuts) 28

Figure G2. Percent deviations from the 2010 indexed price for livestock products for all GCM simulations and decades 29

Figure G3. Deviations from the 2010 indexed price for millet and groundnuts over all GCM simulations in 2050 from GLOBIOM 30

Figure G3. Indexed per capita demand for crop and livestock products (2010=1) for CCAFS scenarios in 2050. 31

H. Development outside West Africa 32

I. Vulnerability and Challenges to adaptation 33

Table I1. Vulnerability indicators and ranking of indicators CCAFS scenario from low to high vulnerability, with factors categorized according to the dimensions from Füssel (2007) 33

J. Examples of the use of the CCAFS West-Africa scenarios 34

References 36

A. Development of the CCAFS scenarios

Scenario development

Regional stakeholders outlined four scenarios, structured along two axes of uncertainty, using narrative flowcharts, conceptual maps, storylines, and a range of semi-quantitative indicators including information on governance, agriculture, food security and livelihoods.

Participants of the CCAFS scenario development workshops

They included representatives from the Economic Community of West African States (ECOWAS), the West and Central African Council for Agricultural Research and Development (CORAF/WECARD), the Permanent Interstate Committee for Drought Control in the Sahel (CILSS, part of ECOWAS), AGRHYMET, a specialized institute of the Permanent Interstate Committee for Drought Control in the Sahel (CILSS) composed of nine member states (Benin, Burkina Faso, Cabo Verde, Côte d'Ivoire, the Gambia, Guinea, Guinea-Bissau, Mali, Mauritania, Niger, Senegal, Chad, and Togo), Institut du Sahel (INSAH), which coordinates exchange of national research on agriculture, population and development issues between its member states, Enda Third World (ENDA-TM), and the Network of Farmers' and Agricultural Producers' Organizations of West Africa (ROPPA) .

Participants represented Senegal (33), Mali (14), Niger (9), Burkina Faso (7), Ghana (5), Nigeria (1), The Gambia (1), and international organizations (13).

For stakeholders, the actors driving change in the region (state and non-state actors) and the time priority of policies (short-term and long-term) were the most uncertain but relevant factors for agriculture and food security in the future. Participants considered non-state actors as the private sector and CSOs. Figure A1 presents short summaries of narratives created by stakeholders.

Facilitators from CCAFS, organizers from Agriculture and Rural Development office of the Economic Community of West African States (ECOWAS), and from the West African Council for Agricultural Research and Development (CORAF/WECARD) guided the process and provided tools for the stakeholders to envision the scenarios. Modelers from IIASA and IFPRI (the institutes where the models used for the quantification are based) were also involved in the process. While they provided insights into what kinds of indicators could be then used as model drivers, their primary role was to gain understanding of the logic and magnitude of the trend indicators which would then guide the translation of the scenario narratives to the quantitative scenario drivers.

State Actors Dominate

Short-term Priorities Dominate

Long-term Priorities Dominate

Cash, Control, and Calories

Governments playing a strong role in governing West Africa's food security and livelihoods, however, short-termism drives government policies. Governments are more focused on urban social stability and security than rural lives. Quick fixes, and fast gains and cash get priority. Quantity is emphasized before quality. The disregard of rural food security eventually leads to increases in the need for food aid and external safety nets such as urban to rural cash flows. Governments become very adept at mobilizing foreign aid money. Commercial, monoculture agriculture is implemented widely leading to environmental degradation and conflicts between agriculturalists and pastoralists. Resource mining for quick food production has destructive long-term effects. Regional integration plans do not last, and the lack of regional policies means that water conflicts occur regularly. On the other hand, vigorous efforts are made to follow the Millennium Development Goals through mass education and decentralization of power.

Self-Determination

Governments, emerging out of a period of uncertainty to relative stability, drive the change through regional collaboration, better tools for effective government and a focus on longer-term investment into infrastructure and access to markets for rural populations, education and direct investments into agriculture. All of this has to be done on a small budget because donor funds have declined after the region's drive to self-determination has resulted in international disputes about outside influence. A measure of regional food self-sufficiency has been achieved by West African countries. However, agricultural intensification has a negative impact on rural employment. Also, increased agricultural productivity and extended land use have impacts on water availability and quality which produces challenges for the region's developments.

Save Yourself

Non-state actors are the driving force of change, governments are passive, corrupt and unstable, playing a facilitating role for the short-term oriented, extractive actions of the private sector. Civil society organizations focus almost exclusively on emergency issues and longer-term development objectives are not part of societal debates. Extra-regional interventions to try and stabilize Mali have failed and instead led to great regional unrest. Hyper-liberal market policies have led to an increasing diversity of available food for the urban middle class, while at the same time the rural poor are highly food insecure due to the fiercely expansive presence of commercial agriculture. Rural livelihoods are decreasing and there are massive movements to urban areas in search of work, ungoverned by national governments. Environmental health has suffered greatly from a lack of policy in this domain and the scramble for new rural sources of livelihood.

Civil Society to the Rescue?

Active private sector interests aiming for the large-scale commercial development of West Africa vie for influence with vibrant and powerful civil society organizations and NGOs who focus on a more community-oriented, sustainable future. This powerful civil society and the private sector collaborate as well as compete for influence, often for the better, for instance contributing to improved livelihoods and knowledge for rural communities. Gender relations have changed and amid the other tensions this transition has been a challenging one. Food security on the whole has improved through a combination of commercial investment in regional food systems which have raised urban food security and an increasing professionalization of relatively small-scale farmers. However, uncertainty around the control of land and resources has threatened the stability of incomes for rural communities.

Non-State Actors Dominate

Figure A1. Short narrative descriptions of the scenarios along the axes of uncertainty: a) will short-term priorities or long-term priorities be the focus of governance and b) will state actors or non-state actors be the driving force in the region?

B. Comparison of the CCAFS Regional Indicators with the SSP Indicators

To add consistency and comprehensiveness to the scenarios themselves, as well as provide a first link between the qualitative narratives and the quantitative modelling, stakeholders provided insights in the form of trend indicators (found in Table B1). Stakeholders provided direction and magnitude of change (given as + and -) and logic behind the change over several time periods. We have separated the indicators are separated into the three major indicator category groups from the SSPs (O'Neill et al., 2015). Semi-quantitative information allows stakeholders to capture the direction and magnitude of change for important indicators of interest to food security, environment and livelihoods. GLOBIOM and IMPACT included many such semi-quantified indicators, translated first into values and used as model inputs, such as regional economic growth, and others that were examined later in the context of model outputs, such as cropland area expansion. This meant that the semi-quantification process allowed the translation of the scenarios to model inputs, but also allowed for a comparison between model outputs and scenarios assumptions.

Table B1. Trend indicators of interest for the CCAFS scenarios

**Demographics/Human Development**	**Economy & Lifestyle/ Policies & Institutions**	**Environment and Natural Resources/ Technology**
Population Growth/Urbanization+, *	Gross Domestic Product+, *	Terrestrial species biodiversity indicator+, *
Women with higher education*	Percent population in poverty*	Marine species biodiversity indicator *
Access to health care*	Dietary Diversity++, *	Forest cover++, *
Access to potable water*	Farmer input prices+	Yields for rainfed crops+, *
Equity*	Transportation infrastructure	Yields for irrigated crops+, *
Prevalence of malaria*	Existence of social protection schemes; percent population covered*	Area for rainfed arable land++, *
	Number of community based organizations *	Area for irrigated arable land++, *
	Corruption index *	Livestock yield change+, *
	Crime rates	Livestock numbers++
	Reports of contaminated food/ food borne diseases; aflatoxins *	Water Availability for Agriculture+, *

+ indicator was translated from semi-quantitative information into values and used as a model input
++ indicator was evaluated in the context of model outputs
** indicates that this indicator aligns to a qualitative element of the SSPs* (O'Neill et al., 2015)

We have mapped further mapped as many SSP indicators to the CCAFS indicators as possible (Table B2). While most CCAFS indicators have only one matching SSP indicator, some CCAFS indicators such as "population growth/urbanization" can be compared to three SSP indicators: "population growth," "urbanization level," and "urbanization type."

Table B2. Mapping of CCAFS semi-quantitative indicators and SSP indicators.

CCAFS Semi-Quantitative Indicators	Demographics<br>Human Development	Economy & Lifestyle<br>Policies & Institutions	Environmental & Natural Resources<br>Technology
Population Growth/Urbanization	Population growth		
	Urbanization Level		
	Urbanization Type		
Women with higher education	Education		
	Gender equality		
Access to health care	Access to health facilities, water, sanitation		
Access to potable water	Access to health facilities, water, sanitation		
Equity	Equity		
Prevalence of malaria	Health investments		
	Access to health facilities, water, sanitation		
Gross Domestic Product		Growth (per capita)	
Percent population in poverty		Inequality	
Dietary diversity		Consumption and Diet	
Farmer input prices		International Trade	Agriculture
Transportation infrastructure		International Trade	
		Institutions	
Existence of social protection schemes; percent population covered		Institutions	
Number of community based organizations	Societal Participation	Institutions	
Corruption index		Institutions	
Crime rates		Institutions	
Reports of contaminated food/ food borne diseases; aflatoxins	Access to health facilities, water, sanitation	Institutions	
Terrestrial species biodiversity indicator		Environmental policy	Environment
Marine species biodiversity indicator		Environmental policy	Environment
Forest cover			Land Use
Livestock yields and numbers			Agriculture
Yields for rainfed/irrigated crops			Agriculture
Area for rainfed/irrigated arable land			Agriculture
			Land Use
water availability for agriculture			Agriculture
			Land Use
			Environment

Note: The first column presents the CCAFS semi-quantitative indicators. The second, third, and fourth columns present the SSP indicators that can be linked to the CCAFS semi-quantitative indicators. Each column represents a separate grouping of indicators from O'Neill et al. (2015): Demographics and Human Development; Economy and Lifestyle and Policies and Institutions; Environmental and Natural Resources and Technology. Colors have been added for the SSP indictors that map to multiple CCAFS indicators.

In the Table B3, we provide an example of mapping the CCAFS semi-quantitative indicators to the SSP indicators using the semi-quantitative information from the stakeholders developed in the workshops and the qualitative information on the SSPs from O'Neill et al. (2015). We map the CCAFS indicator "access to health care", "access to potable water", and "prevalence of malaria" to the under the "access to health facilities, water, and sanitation" indicator from the narrative elaboration of the SSPs (O'Neill et al., 2015), named in the first column. The CCAFS scenario is given in the second column. Column 8 contains the SSP name that corresponds 1 to 1 with the CCAFS scenario and the SSP indicator to compare is in Column 9 and 11. The qualitative information for each indicator of each SSP is taken from O'Neill et al. (2015) and appears in Column 10 and 12. We have added color to the qualitative information of each indicator based on how well the narrative/indicator of each SSP fits with the narrative/indicator of each CCAFS scenario. Green indicates a "good match", yellow indicates a "neutral match", and red indicates a "bad match".

For *Save Yourself*, *SSP3: Fragmentation* matches well in that both scenarios assume low access to health care, however access to potable water improves, though not dramatically, due to profit-driven investments, which is not in line with SSP qualitative information. *Self-Determination* and *Civil Society to the Rescue*? also match to their SSP well as they both describe improvements to the access to health care and potable water, high (*SSP1: Sustainability*) and medium (*SSP2: Middle of the Road*) access, respectively. However, *Cash, Control, Calories* does not map as well because the CCAFS narrative describes an improving, but unstable future where some strides are made to improve access but the system is still overwhelmed by the growing population, whereas *SSP 5: Conventional Development* sees a future with high access to health care and sanitation. We have flagged this mis-mapping as "neutral", because it neither supports a good match to the SSP nor supports a bad match to this SSP.

The scenario insights into the prevalence of malaria in the region are complicated to map directly to the SSP indicators for access to health care and sanitation because prevention of malaria relies on not only investments in water and sanitation infrastructure to limit mosquito breeding, but also access to and use of preventative measures such as mosquito nets. In *Civil Society to the Rescue?*, action to reduce the prevalence of malaria begins quickly and continues over the entire period due to the actions of the CSOs in deploying mosquito nets, an effective action that cannot be captured within the SSP indicators, while in *Self-Determination* actions by the state to improve water infrastructures and sanitation improve the incidence, albeit later in the period. We view the mis-mappings of the SSP indicators for health investments and access to health care and sanitation with CCAFS scenario indicator for malaria as an important message from the regional stakeholders that improving livelihoods requires engagement from multiple institutions as well as scientific and culture understanding of the multiple challenges to and solutions for a problem as complicated as the prevention and treatment of malaria.

CCAFS Ind.	CCAFS Scenario	2010-2020	2020-2030	Logic	2030-2050	Logic	SSP	SSP Ind.	SSP qual. info.	SSP Ind.	SSP qual. Info.
Prevalence of malaria	Cash, Control, Calories	+	+	Shame related to disease - sanitation issues due to increasing population	+	Governments are able to cure but not prevent	SSP5	Access to health facilities, water, sanitation	High	Health investments	High
	Self-Determination	+	+	No change at first	--	Vaccine supported by government policy; education; better water infrastructure; sanitation	SSP1		High		High
	Civil Society to the Rescue?	-	-	easily deployable technology by CSOs (impregnated mosquito nets etc.)	---	Vaccine available - but not for everybody	SSP2		Medium		Medium
	Save Yourself	++	++	Moderated by non-state, environment-borne diseases, lack of infrastructure/facilities to reach sick	++	Civil society slightly better at dealing with outbreaks but largely continues	SSP3		Low		Low
Access to health care	Cash, Control, Calories	=	+	Government and private sector investments are limited, knowledge capacity limited, slow, quality questionable	+	Trend continues; population overwhelms available health care; capacity to deal with some emergencies does exist; governments can leverage donor funds well.	SSP5	Access to health facilities, water, sanitation	High		
	Self-Determination	+	+	Slow improvement through government policies	++	Institutional, knowledge and technical capacity have developed	SSP1		High		
	Civil Society to the Rescue?	=	+	CSOs not best equipped to deploy health care infrastructure; private sector invests but not available for the poorest	+	Technology increases health care for middle and upper classes, but not for poorest - tech still costs money	SSP2		Medium		
	Save Yourself	-	-	Prices too high, street medicine, fake medicaments	-	New technologies available but these do not reach the majority	SSP3		Low		
Access to potable water	Cash, Control, Calories	+	+	Government investment, focusing on a short-term issue; debate for privatization will be high	=	Failure of long-term insight into population growth	SSP5	Access to health facilities, water, sanitation	High		
	Self-Determination	+	++	Access for whom? Government willingness, but limiting factors are technology, pollution	++	Investment in infrastructure; pressure from population; pollution by irrigated agriculture; climate change	SSP1		High		
	Civil Society to the Rescue?	-	+	Private and civil society intervention and collaboration	+	Technology increases, but population pressures and CC impacts also increase	SSP2		Medium		
	Save Yourself	=	+	Some investment when profits can be made	+	Water is available when profitable	SSP3		Low		

Table B3. Comparison of a selection of the CCAFS indicators with the SSP indicators using the qualitative information, logic, and trends

C. Model Comparison

GLOBIOM and IMPACT are partial equilibrium models with a significant focus on the agriculture sector. Both models have varying degrees of spatial disaggregation and consider the demand and use of products for food, livestock feed, bioenergy, and process production (Havlík et al., 2011; Robinson et al., 2015). Both models employ a double-log demand system to model consumer food demand, considering both a dynamic adjustment to demand based on income growth as well as a demand response based on prices (Valin et al. 2014). The features of both models are presented in Table C1.

Table C1. GLOBIOM and IMPACT comparison

	GLOBIOM	IMPACT
**Economic Sector**	Agriculture sector including crops, livestock and grasslands, bioenergy, and forestry	Agriculture sector including crops, ag. processing, and livestock
**Time Horizon**	2000–2030/2050/2100	2005-2050
**Role of Markets**	Regional markets linked through global markets determine supply and demand	Global markets determine supply and demand
**Geography**	Global representing 30 country/regions	Global representing 159 country/regions
**Resolution of Production side**	Bottom-up approach at detailed grid-cell level (>10,000 worldwide) (4 crop production systems and 8 livestock production systems)	320 food production units (intersection of national and hydrological boundaries) (2 crop production systems and 8 livestock production systems)
**Commodities**	18 crops, 7 livestock products, 5 forest products, 9 bioenergy products	39 crops, 6 livestock products, 15 processed goods
**Environment**	GHG accounting, irrigation water use, and endogenous land-use and land cover change	Hydrology, water basin management of irrigation water, exogenous and endogenous cropland area expansion
**Climate Change Impact**	EPIC crop model	DSSAT crop models and linked hydrology models

D. Model Drivers

Cropland area expansion

GLOBIOM captures historical and traditional production systems and land use in its base year (2000), which is based on available historical data. Future changes from this starting point are simulated endogenously in the model, with transitions between production systems (i.e. extensive systems to more intensive systems) and land uses (i.e. forest to pasture) determined by changes in their relative profitability. This profitability is determined by changes in the value of products (e.g. crops, livestock product, timber, etc.), the productivity of the economic activities that produce them, as well as the costs of changing production from one system to another (Havlík et al., 2014).

Regional Integration Impact on Farm Input Costs

GLOBIOM includes challenges to regional integration through impacts on farm input costs. The following section offers details on the relative impact on farm input costs.

Competing national interests and standards often clash with competing international donor initiatives (Rohrbach et al., 2003). The state-led regional integration policies of *Cash, Control, and Calories*, are poorly coordinated and lack a long term focus, which over time cause similar failures in regional integration noted by Tarvares and Tang (2013), such as countries overly committed to integration programs in multiple, overlapping regional organizations leading to lower program implementation overall and programs that are overly bureaucratic and lack involvement from the private sector, increasing the costs of production for the region increase by 15%. The weak governments unable to control the regional stability in *Save Yourself*, create an environment where conflicts severely limit access to markets and raise costs of production 25% for farmers. In *Civil Society to the Rescue?*, lack of coordination between the private sector and civil society organizations in negotiating integration programs and investing in infrastructure planning increase the costs of production for farmers (smallholder and large scale), limited to 15%. *Self-Determination* sees improved regional integration through merging of regional organizations led by member countries with a focus on the long-term development of the region and therefore the production costs for the region remain unchanged.

Socioeconomic drivers

Population growth and the economic development of the region will have impacts on future food demand, as well as the region's investments and advancements in agricultural productivity. Figure D1 presents the macroeconomic development of the region within the context of the SSPs. Population growth is shown in Figure D2. The per capita economic growth over the time period was first realized by the stakeholder developed narratives of the scenarios and then given trend behaviors and logic for change along with a series of other indicators. To translate these to numerical values we used "one to one" mapping of SSP to CCAFS scenario and used the trend indicators to adjust the pathways. guided by the regional scenario narratives and the trend indications of change developed by the stakeholders during the scenario development workshops, we adjusted these drivers for the region to capture some of the uncertainty around governance and political stability that are captured by the regional scenarios as they pose a challenge for development in Western Africa (Palazzo et al., 2016; Palazzo et al., 2014)

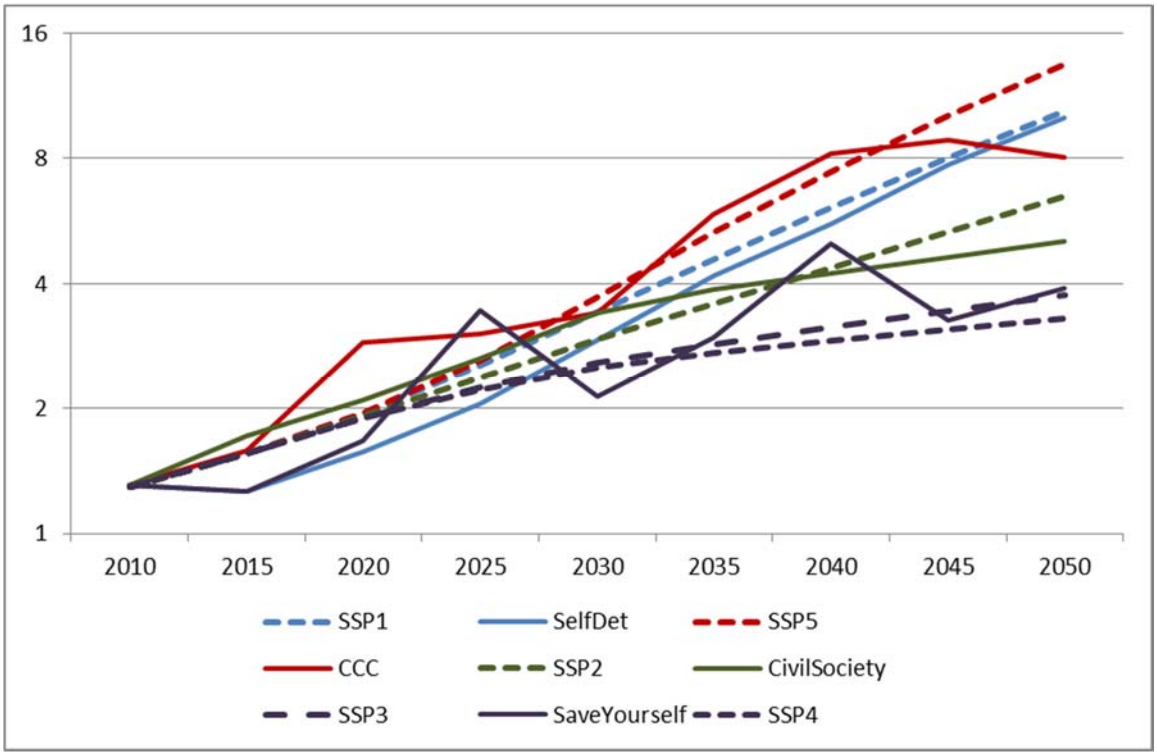

Figure D1: Relative change in GDP per capita for West Africa 2000-2050 for the CCAFS scenarios and SSPs (2000=1) GDP values from OECD (Dellink et al., 2015) and population values from IIASA (Kc and Lutz, 2014)

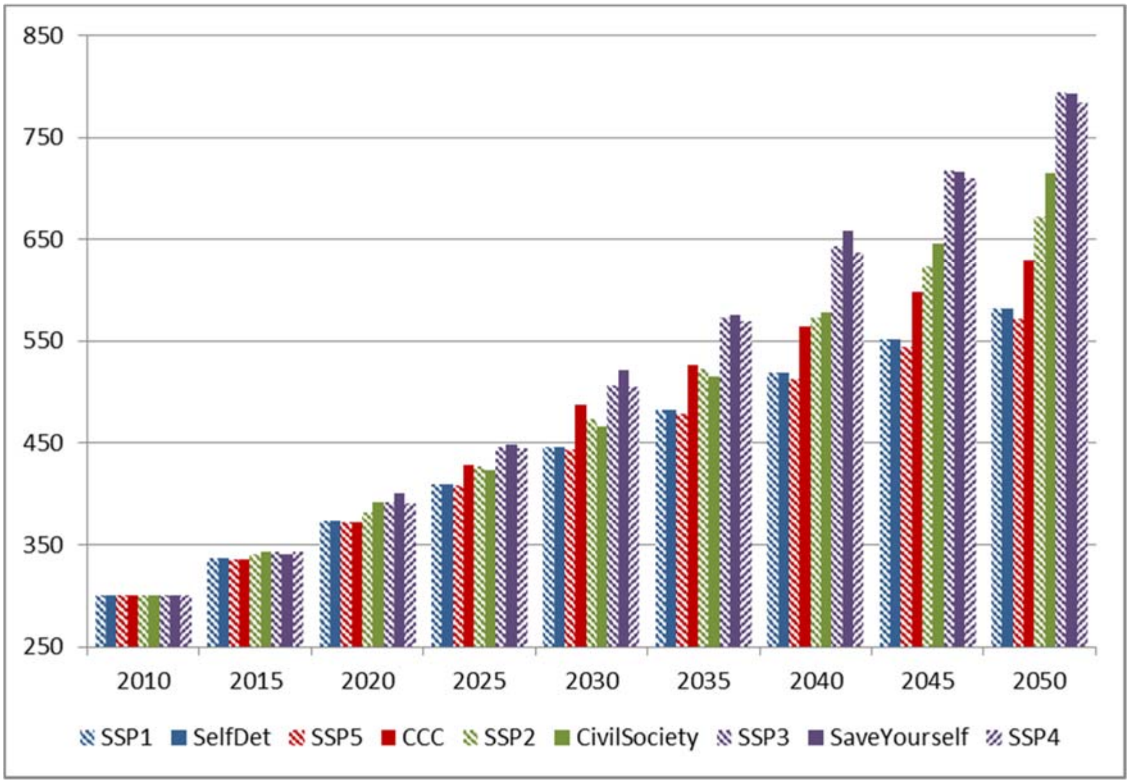

Figure D2: Population of Western Africa in millions of people for the CCAFS scenarios and SSPs (population values from IIASA (Kc and Lutz, 2014))

Note that the axis begins at 250 million.

E. Crop and livestock production and share of global production

Western Africa, as a region, is the leader, or among the top global producers of cassava, millet, and sorghum (FAOSTAT, 2015). In particular, the sorghum, millet, and cassava production in the region accounts for nearly 20, 27, and 41% of the total global production, respectfully.

Figure E1 shows each crop's share of production within West Africa and also within the global context. The large circle represents each crop's share of the total crop production in Western Africa (excluding tree crops) in dry matter. The colors of the slices represent different crops. The smaller circles represent the total global production of three major crops (cassava and other roots and tubers, millet and sorghum). In the smaller circles, the slice that is colorful represents Western Africa's share of the total global production of the respective crops. For example, in 2010, millet accounted 20% of West Africa's total crop production (large circle), which contributes to 41% of the global millet production (small circle). Note that for the 2050 scenarios, Western Africa's share of global production of maize has been removed because the share remains very small.

Although the agricultural sector faces low crop yields, the region produces and will continue to produce a significant share of the global production for a selection of crops. Large shares of these crops are consumed within the region, but trade in these crops continues to be important in the future. Cassava is presently a staple food crop in the region and will continue to serve as a vital crop for the region, both for food consumption and, under changing diet preferences due to increasing incomes, as livestock feed. Even when strides are made to improve productivity, the region's agricultural sector cannot keep up with the growing regional and global demands, and for many crops, competitiveness declines and the region sees an increased share of imports relative to the region's overall production, cassava included. Sustainable intensification with a climate-smart agriculture perspective could be an option to explore (Campbell et al. 2014).

The development of the livestock sector in West Africa, depends on not only the overall productivity in the region to meet the growing demand but also supporting the transformation of livestock systems from pastoral to mixed systems, where more productive livestock both graze and consume feed crops.

In terms of livestock production, the increase in both ruminant and monogastric meat is the largest for Self-Determination (closely followed by Cash, Control, Calories), Save Yourself has the least productive livestock sector of the scenarios, but still the scenario sees huge expansion in the dairy sector. While the per capita demand for dairy is the lowest in Save Yourself, due to lower economic growth, the total dairy demand is the highest following population increase, driving the growth in supply, although most of the demand for dairy is met with imports.

2010

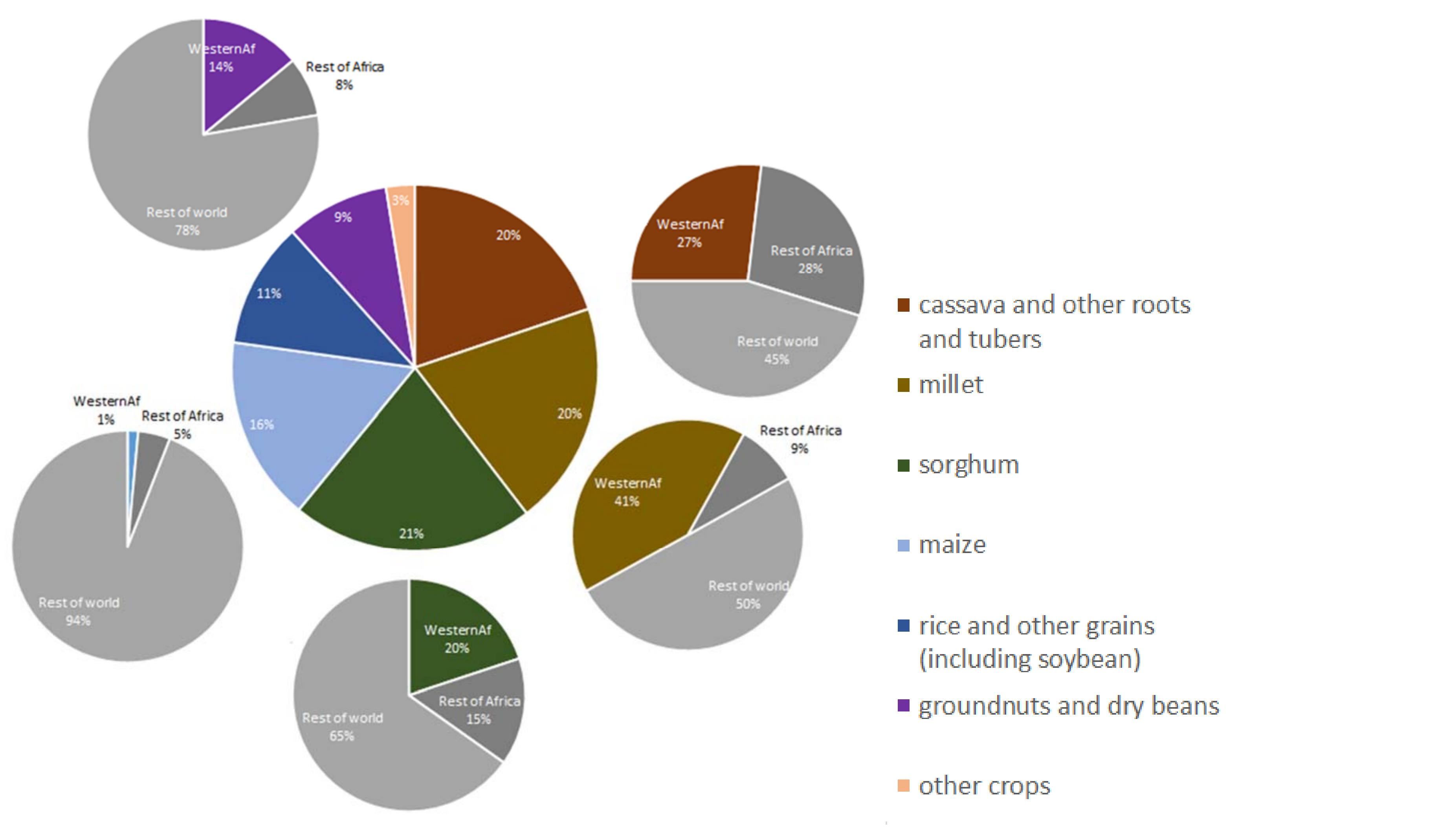
WesternAf
14%
Rest of Africa
8%
Rest of world
78%
3%
9%
20%
11%
16%
20%
21%
WesternAf
27%
Rest of Africa
28%
Rest of world
45%
WesternAf
1%
Rest of Africa
5%
Rest of world
94%
WesternAf
41%
Rest of Africa
9%
Rest of world
50%
WesternAf
20%
Rest of Africa
15%
Rest of world
65%
cassava and other roots and tubers
millet
sorghum
maize
rice and other grains (including soybean)
groundnuts and dry beans
other crops

Self Determination in 2050

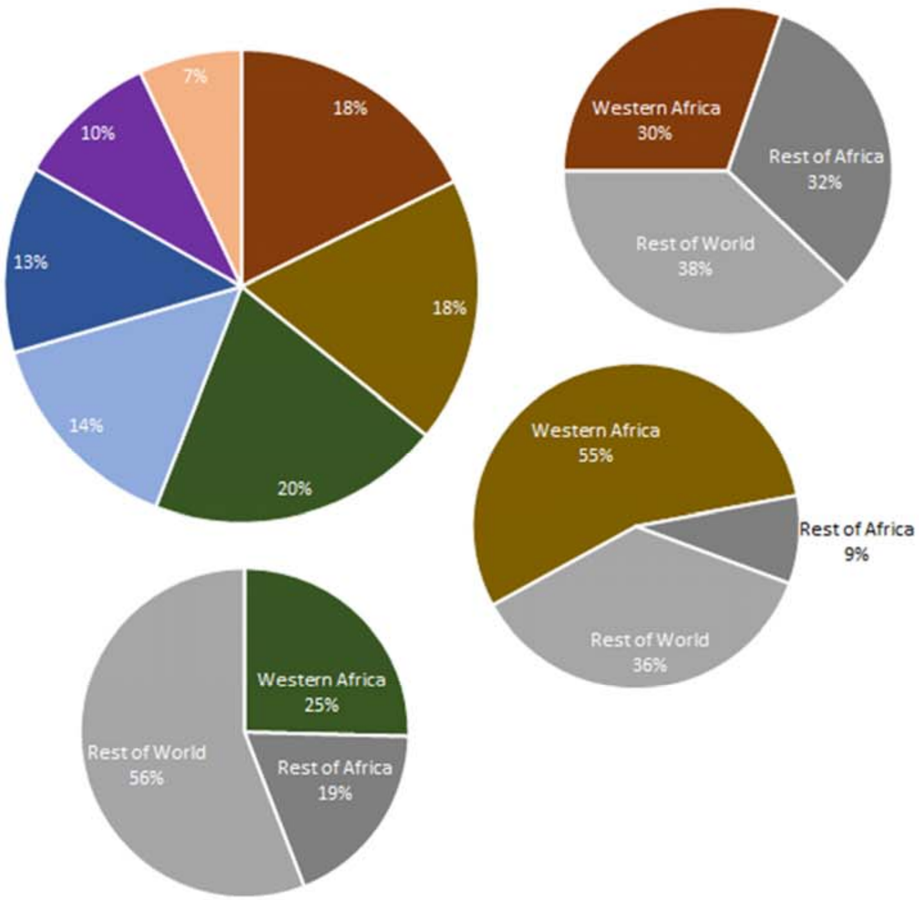

Cash, Control, and Calories in 2050

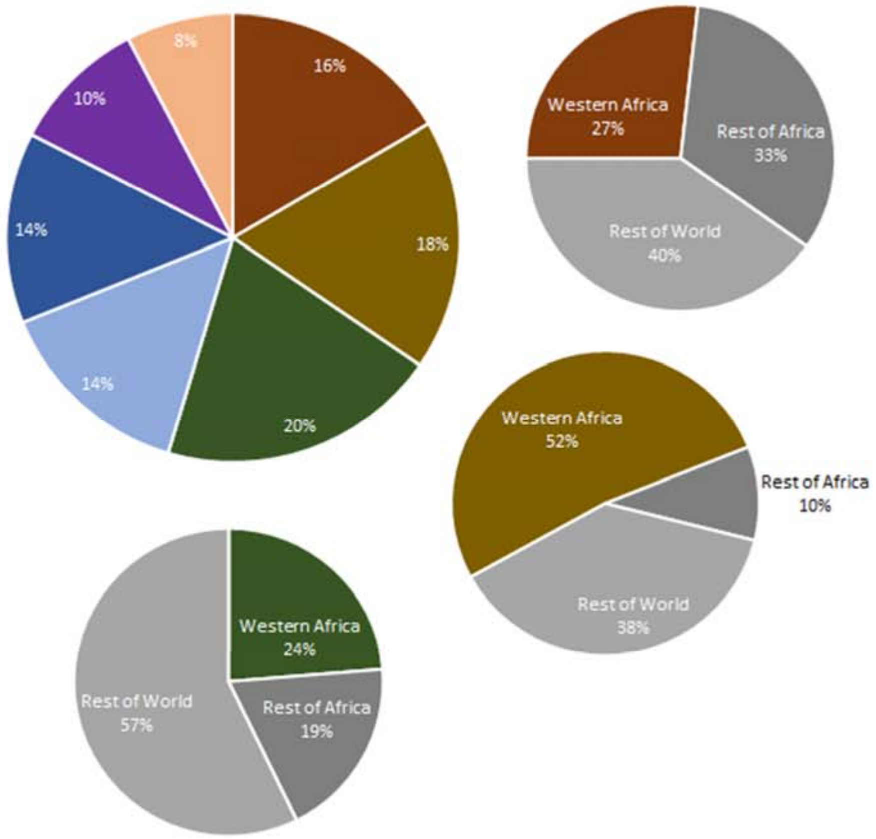

Civil Society to the Rescue in 2050	Save Yourself in 2050
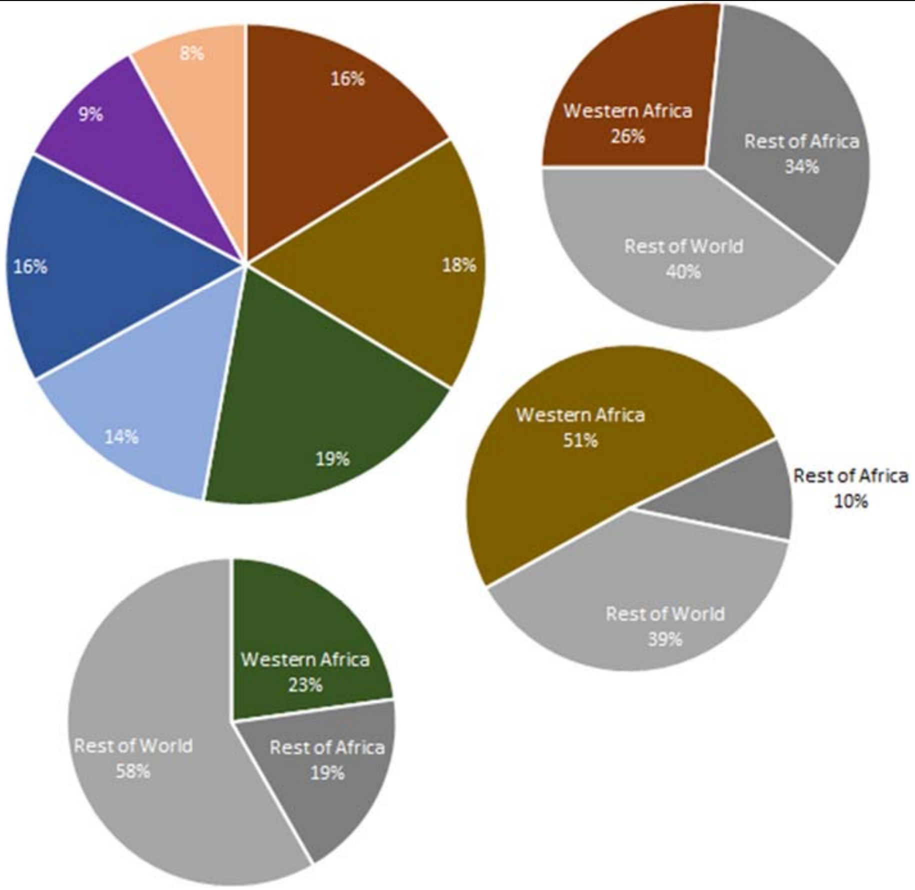	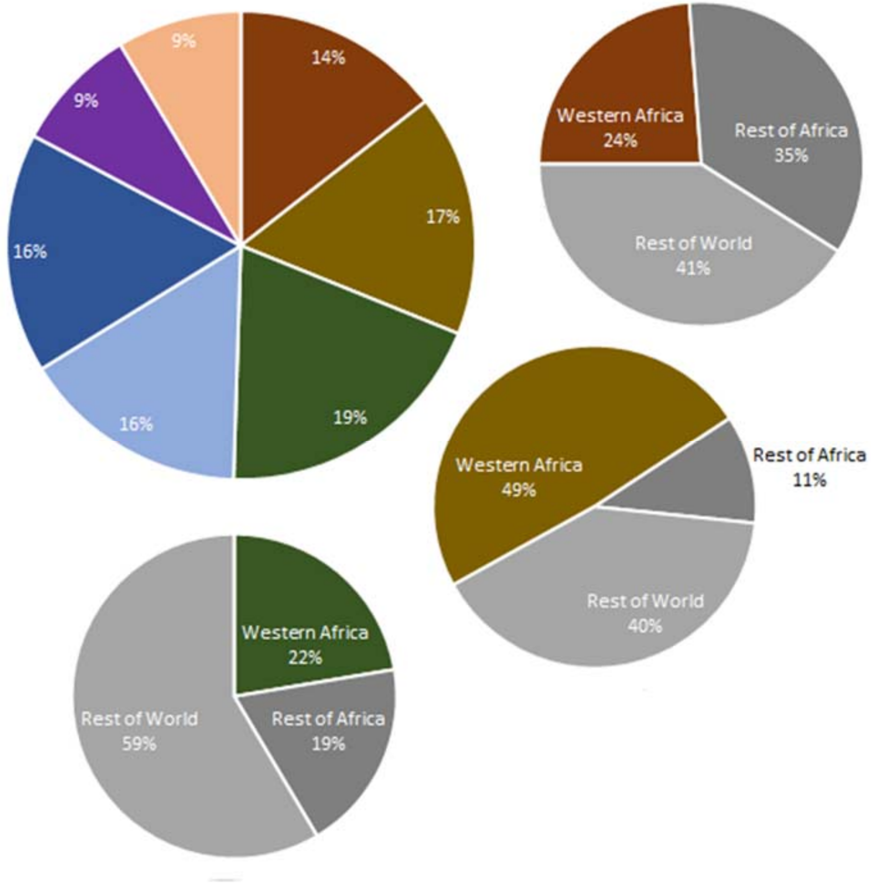

Figure E1. Western Africa's share of crop production by crop (large circles) and the region's production share of global production by crop (smaller circles) in dry matter for 2010 (top most figure) and in 2050 by scenario (figures in lower four rectangles) Source: FAOSTAT 2015 (2010); GLOBIOM model results (2050).

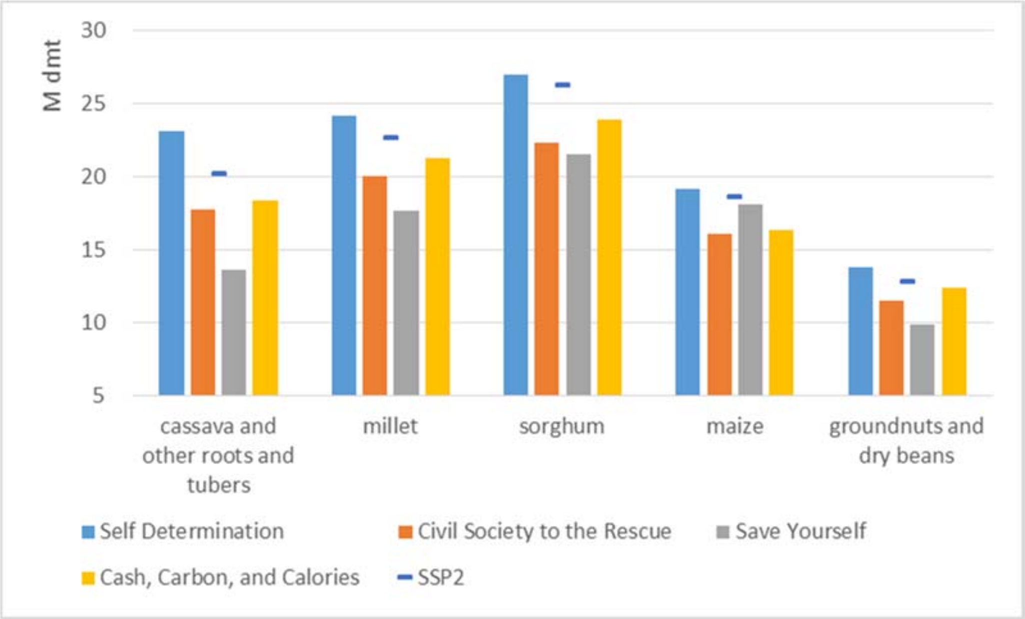

Figure E2. Growth in production of selected crops in Western Africa by CCAFS scenario and SSP2 from 2010 to 2050 in million dry matter tons from GLOBIOM

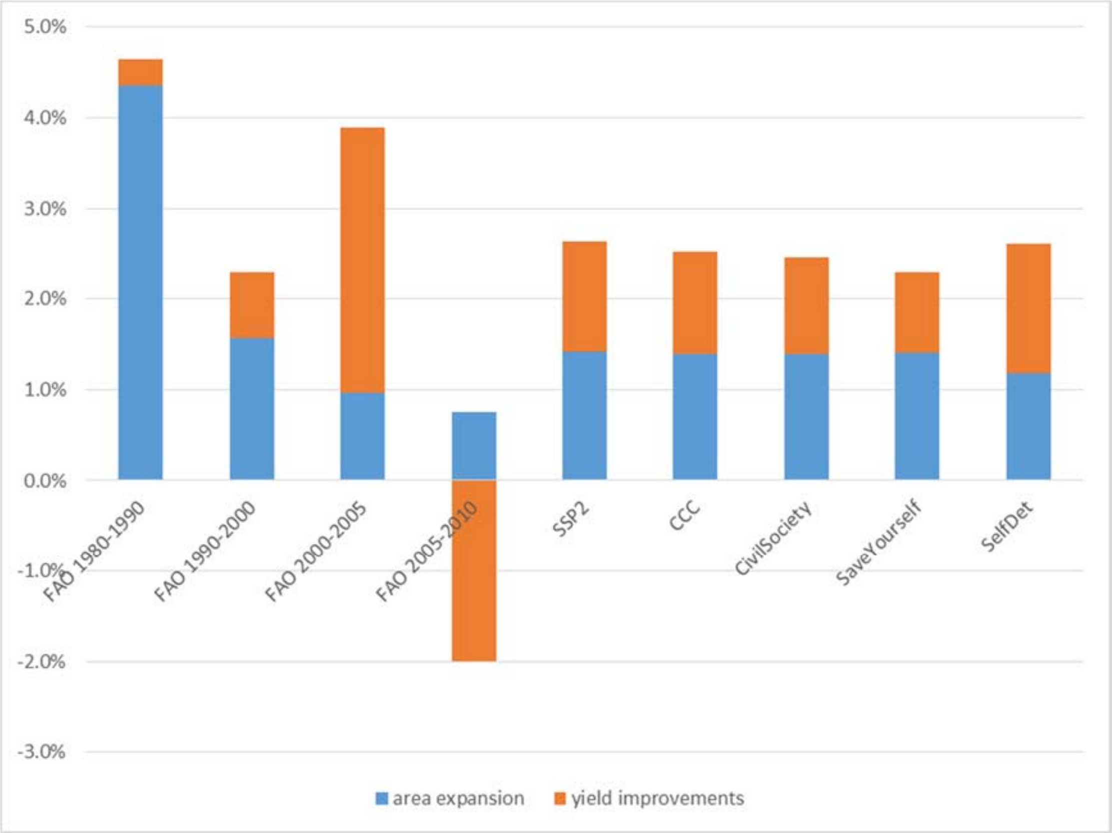

Figure E3. Millet average annual growth in production from FAO statistics (FAOSTAT, 2015) and CCAFS scenarios from 2010-2050 the growth in production is allocated to either expansion of area or improvement in yields

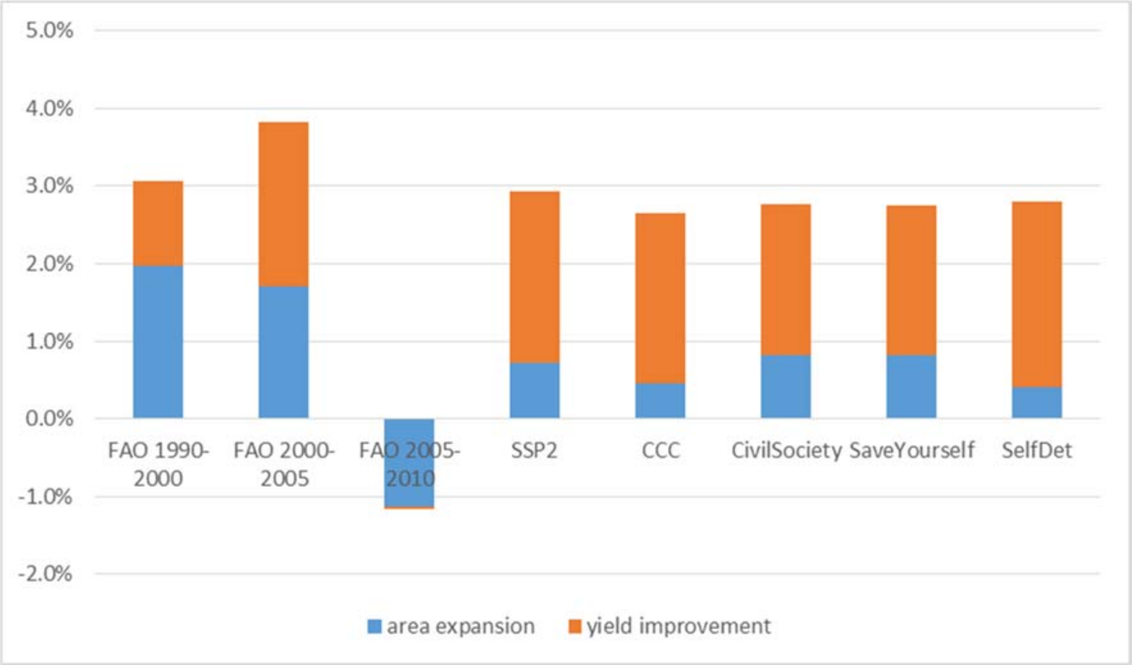

Figure E4. Sorghum average annual growth in production from FAO statistics (FAOSTAT, 2015) and CCAFS scenarios from 2010-2050 the growth in production is allocated to either expansion of area or improvement in yields

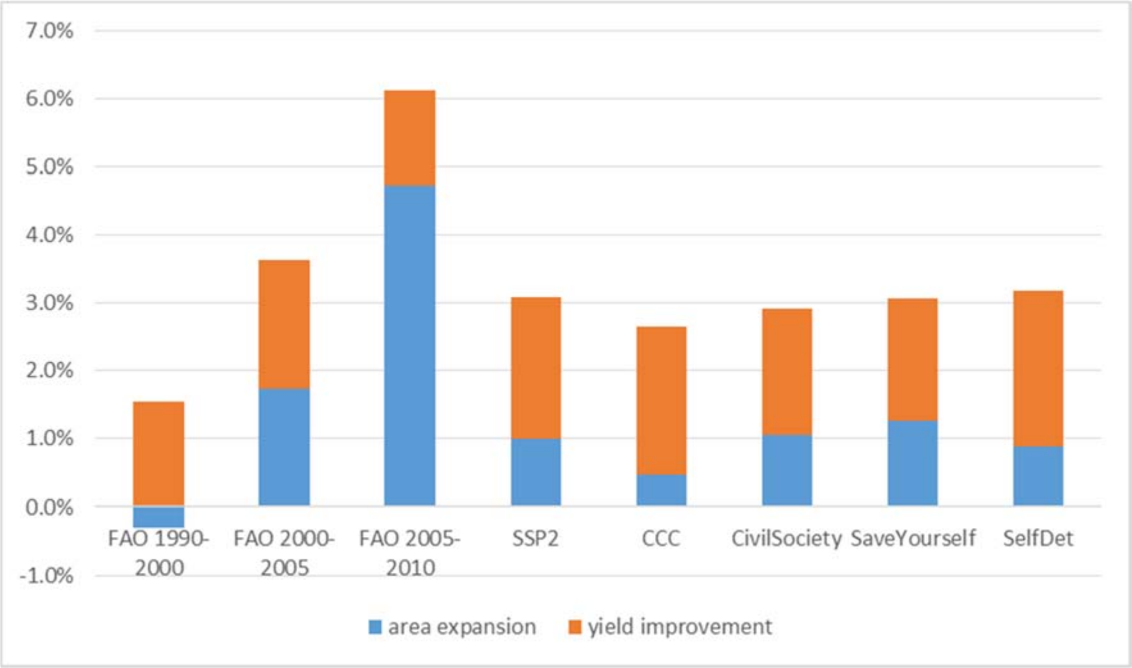

Figure E5. Maize average annual growth in production from FAO statistics (FAOSTAT, 2015) and CCAFS scenarios from 2010-2050 the growth in production is allocated to either expansion of area or improvement in yields

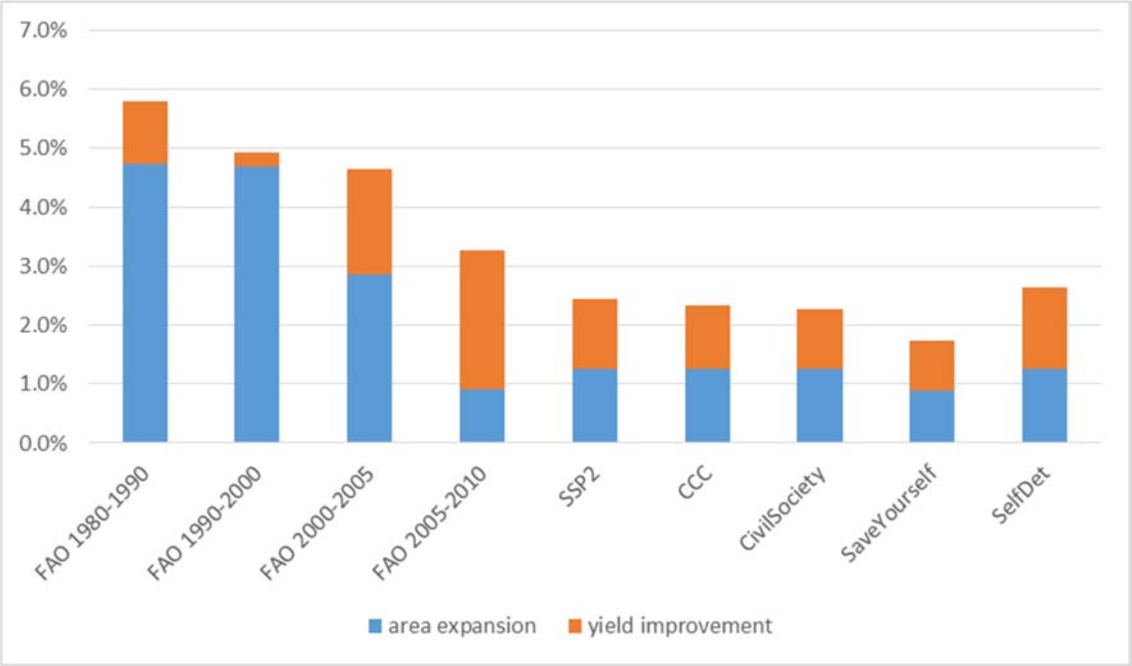

Figure E6. Cassava average annual growth in production from FAO statistics (FAOSTAT, 2015) and CCAFS scenarios from 2010-2050 the growth in production is allocated to either expansion of area or improvement in yields

F. Applied Climate Change impacts in GLOBIOM and IMPACT

Climate change is considered to be a major challenge for food security but the degree of climate change and scale of its impacts was not limited to any one scenario but considered as an outside driver in all scenarios as it is outside the decision context for the region, similar to the way in which climate change impacts are considered within the climate change community has considered impacts when developing scenarios for within the SSP framework.

Additionally, assessments of agriculture under future climate change have considered impacts of alternative adaptation strategies (Leclère et al., 2014) and the role of trade (Mosnier et al., 2014) using the GLOBIOM model. Expanding on the global climate and adaptation analysis of agriculture from Nelson et al (2010), which uses the IMPACT model to examine the effects of climate change under three global socio-economic futures, national level impacts the focus for three regions within Africa (Hachigonta et al., 2013; Jalloh et al., 2013; Waithaka et al., 2013).

IMPACT and GLOBIOM consider multiple general circulation models (GCMs) and different biophysical crop models to capture the uncertainty of the biophysical impacts of climate change on crop yields (Table F1). Impacts are not limited to only the Western African region but applied globally using the relative changes in the globally-gridded crop models yields from 2000 (Nelson et al., 2010; 2014a, 2014b; von Lampe et al., 2014). However, within IMPACT, total cropland area is considered an exogenous driver and the endogenous response to climate impacts is limited to the reallocation of area among the individual crop types.

Table F1 Climate Scenarios

Representative Concentration Pathway	General Circulation Models	Crop Model Suite[1]	Crops[2]	Grassland	CO2 Fertilization
8.5	GFDL-ESM2M<br>HadGEM2-ES<br>IPSL-CM5<br>MIROC-ESM	EPIC	Barley, Dry Beans, Cassava, Chickpea, Maize, Cotton, Groundnut, Millet, Potato, Rapeseed, Rice, Soybeans, Sorghum, Sugarcane, Sunflower, Sweet Potato, and Wheat	Yes	Additional CO2 fertilization for both crops and grassland
		DSSAT	Groundnut, Maize, Potato, Rice, Sorghum, Soybean, and Wheat	No	CO2 fertilization at current levels
Constant 2000 climate	Represented by each model's baseline assumptions without climate change				

Notes: All GCM climate data comes from CMIP and ISI MIP (Taylor et al. 2012) and are downscaled for use in the crop models.

[1] *The EPIC crop models are used by GLOBIOM, and the DSSAT crop models are used by IMPACT*

[2] *Table F.1 in Robinson et al.* (2015) *provides the most detailed mapping based on biophysical similarities of the crops represented in IMPACT but not covered by the crop model DSSAT*

The modeling community has not evaluated the climate effects on grassland productivity to the same extent as crop productivity (Wheeler and Reynolds, 2012). In GLOBIOM these effects are now used to understand the economic and land use implications (Havlík et al., 2015). The strictly biophysical climate impacts on grassland productivity as modeled by EPIC and are relatively negative for the region (Figure F1**Error! Reference source not found.**). In Figure 1, each colored circle represents the difference in grassland production relative to the 2050 no climate change value for each scenario. Climate change for all GCMs except MIROC, reduces grassland production. Although the quantification of the drivers for the CCAFS scenarios did not consider direct impacts on grassland productivity, the feedbacks from other drivers as well as expansion of grassland for livestock production increase Western Africa's grassland production compared to the climate change future in all scenarios except for Save Yourself. The overall production in *Save Yourself* is less than the potential production under climate change with no endogenous feedbacks, suggesting that actions or inactions this scenario, such as expanding pastures and limited productivity for animal feed, could exacerbate the effects of climate change (Figure F2).

Historically, cassava has been planted in the region because of its hardiness in periods of drought, pest, and disease resistance in an effort to reduce periods of food insecurity (Hillocks, 2002). Despite its hardiness, the impact of climate change on cassava yields is considerable and when compared to a future without climate change, although GLOBIOM and IMPACT differ in the magnitude of the climate impacts due to the nature of the crop models used to simulate the yield effects. For cassava, the impact of climate change on cassava yields is considerable and when compared to a future without climate change, -2%/-20% and +3%/-1%, GLOBIOM and IMPACT respectively (Table F2). IMPACT uses the DSSAT model (Hoogenboom et al., 2012; Jones et al., 2003), where cassava is not represented but instead applies for this crop the same impact as for potato yields. The climate effects on maize are negative for both models though the magnitude of effects is stronger for IMPACT than GLOBIOM. The climate impacts on millet within GLOBIOM may seem inconsistent since they range from -18% to +23% of the no climate change yields in 2050, however, three out of the four GCM models used to model the crop effects find negative climate impacts which is consistent with other assessments under varied climatic conditions using West Africa specific crop models (Sultan et al., 2013). In the crop modeling suite used by IMPACT, climate impacts are limited for millet and sorghum, highlighting perhaps a need for future improvement in the process-based modeling.

Table F2. Crop yields indexed to the 2010 values with the endogenous scenario impacts and climate effects for a selection of six crops by model

GLOBIOM- Cassava

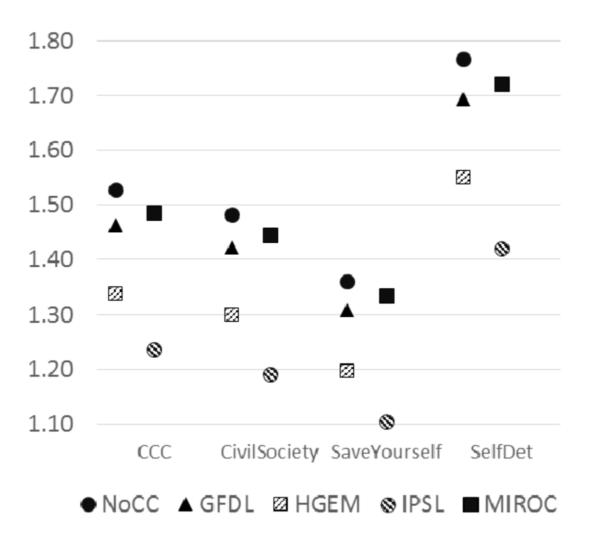

GLOBIOM- Sorghum

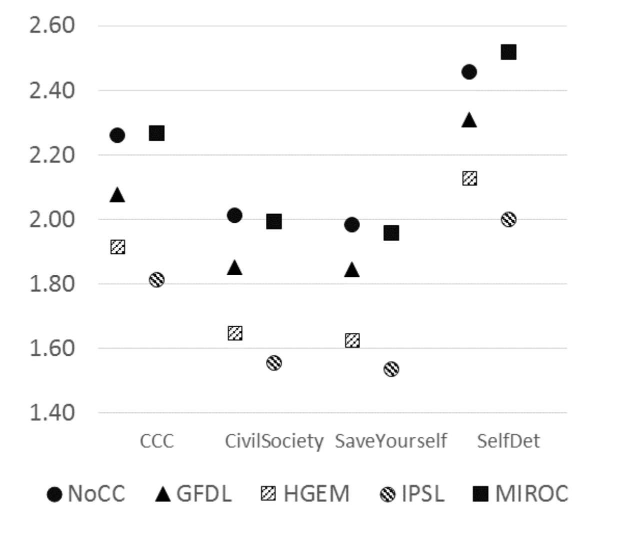

GLOBIOM- Millet

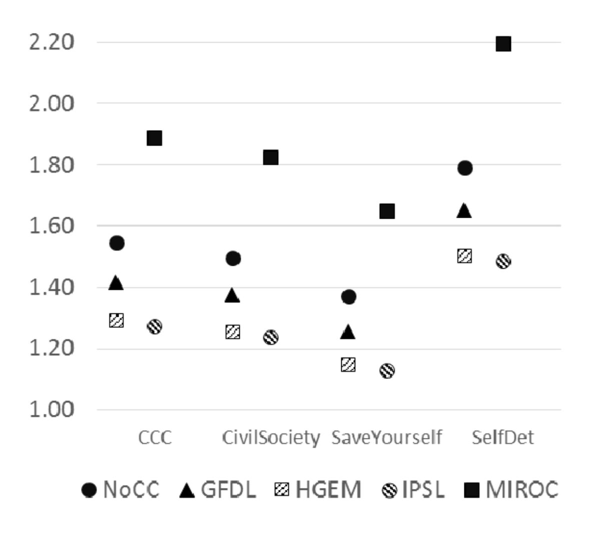

IMPACT- Cassava

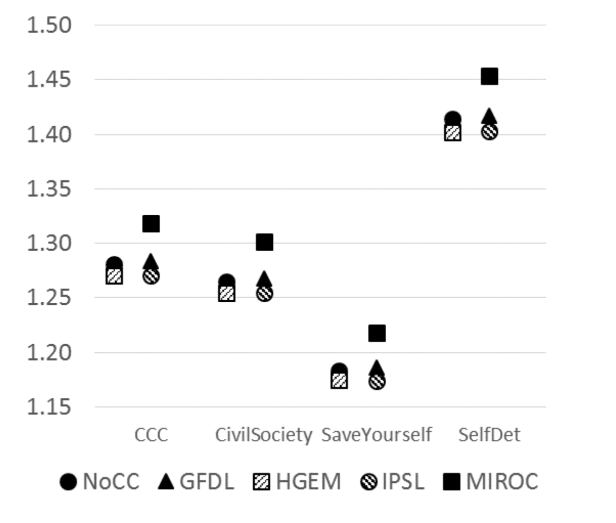

IMPACT- Sorghum

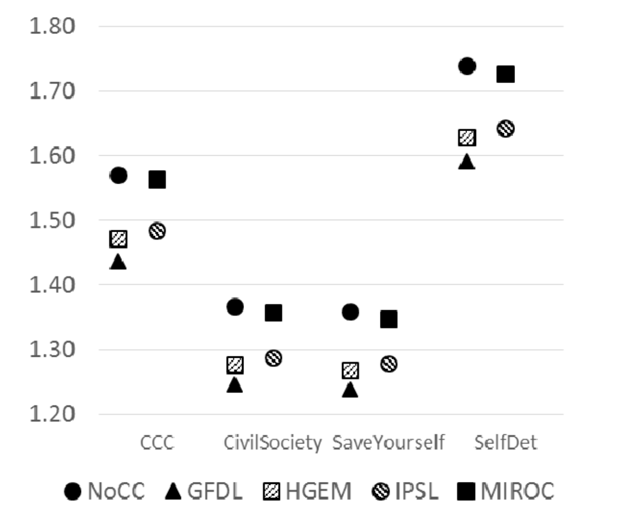

IMPACT- Millet

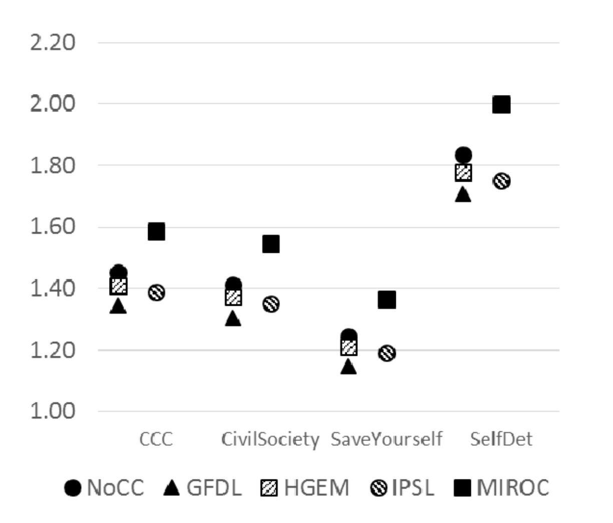

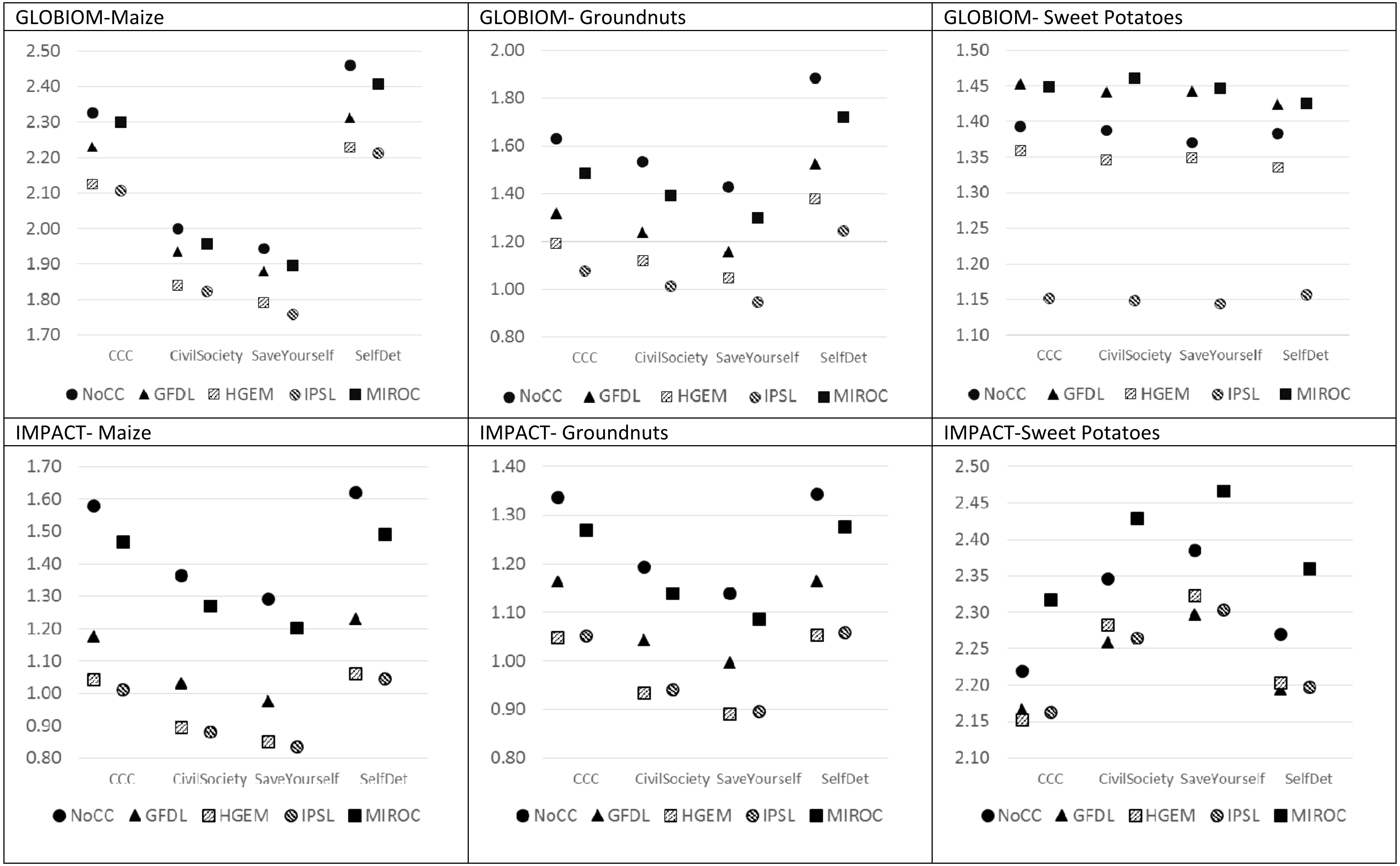
GLOBIOM-Maize
2.50
2.40
2.30
2.20
2.10
2.00
1.90
1.80
1.70
CCC
CivilSociety
SaveYourself
SelfDet
NoCC GFDL HGEM IPSL MIROC
GLOBIOM- Groundnuts
2.00
1.80
1.60
1.40
1.20
1.00
0.80
CCC
CivilSociety
SaveYourself
SelfDet
NoCC GFDL HGEM IPSL MIROC
GLOBIOM- Sweet Potatoes
1.50
1.45
1.40
1.35
1.30
1.25
1.20
1.15
1.10
CCC
CivilSociety
SaveYourself
SelfDet
NoCC GFDL HGEM IPSL MIROC
IMPACT- Maize
1.70
1.60
1.50
1.40
1.30
1.20
1.10
1.00
0.90
0.80
CCC
CivilSociety
SaveYourself
SelfDet
NoCC GFDL HGEM IPSL MIROC
IMPACT- Groundnuts
1.40
1.30
1.20
1.10
1.00
0.90
0.80
CCC
CivilSociety
SaveYourself
SelfDet
NoCC GFDL HGEM IPSL MIROC
IMPACT-Sweet Potatoes
2.50
2.45
2.40
2.35
2.30
2.25
2.20
2.15
2.10
CCC
CivilSociety
SaveYourself
SelfDet
NoCC GFDL HGEM IPSL MIROC

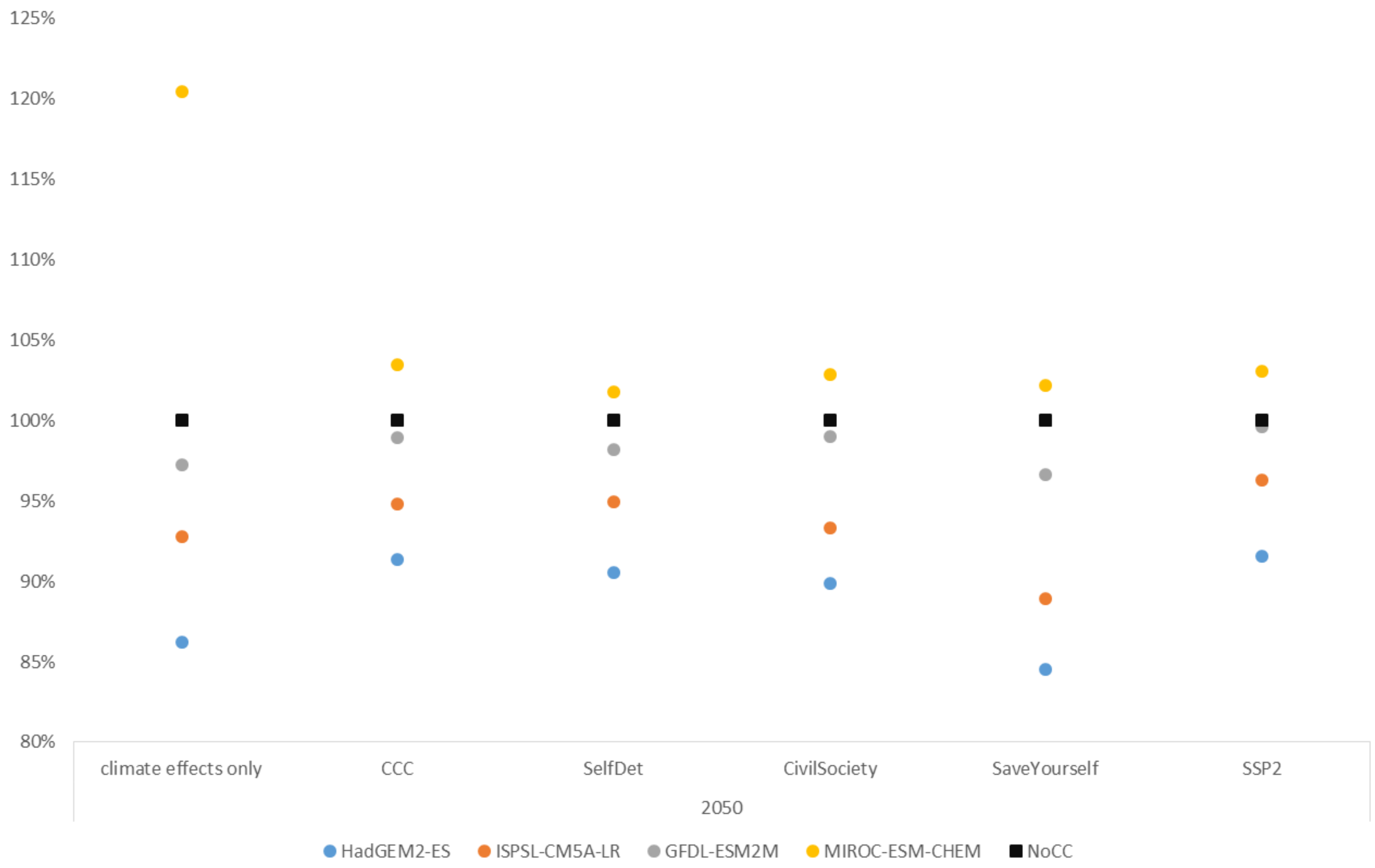

Figure F1. Exogenous biophysical effects of climate change on grassland productivity and endogenous productivity effects under climate change and CCAFS scenarios in GLOBIOM (percent of no climate change 2050 grassland productivity)

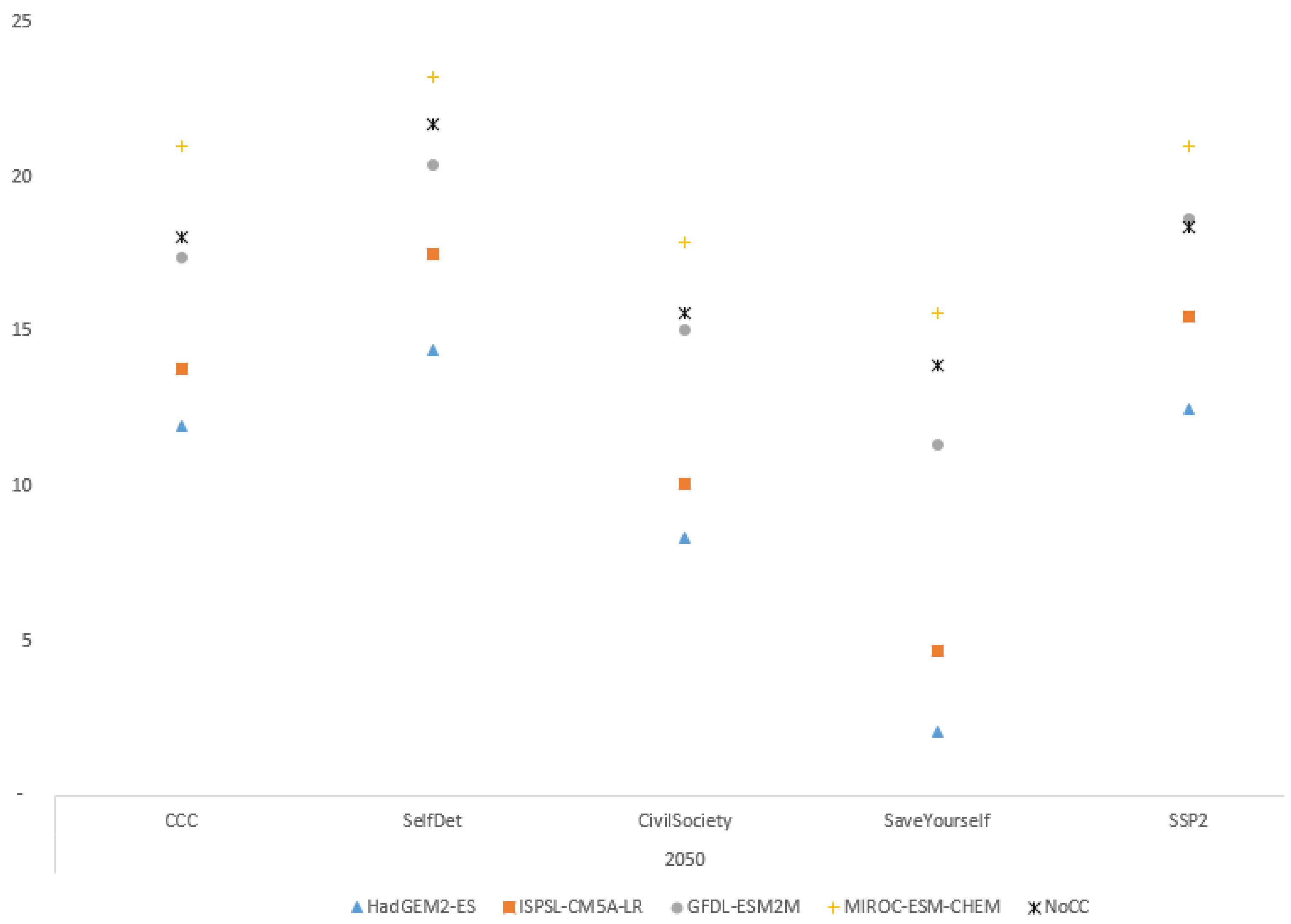

Figure F2. Increase in grassland production from 2010 values by CCAFS scenario and under climate change effects in GLOBIOM (M mt)

G. Per capita demand, prices, and net trade

GLOBIOM considers bilateral trade policies and barriers as well as transportation costs, making the regional prices more responsive to regional effects, whereas IMPACT assumes a perfect price transmission from global markets to regional markets, making prices relatively unresponsive to regional effects. The following results for prices focus only on the GLOBIOM regional prices rather than the IMPACT prices.

By 2050, the average price for crops increases over time for Save Yourself and Civil Society to Rescue, while decreasing for Cash, Control, Calories, and Self-Determination. However, climate change increases the average prices for all crops (for 3 of the 4 GCMs). In 2050, prices under climate change increase additional 15%, on average, in Save Yourself, though only 4% in Self-Determination. Appendix F examines in more detail the variability of prices from GLOBIOM in the scenarios and under climate change

The average regional price for all crops increases over time for both Save Yourself and Civil Society to the Rescue? (+11% and +6% higher in 2050 than in 2010). By 2050, relative prices decrease in Cash, Control, Calories and Self-Determination sees an overall decline (-1% and -5% lower in 2050 than in 2010).

Dairy prices remain relatively stable and by 2050 decrease slightly (-3%) in all the scenarios. Prices for monogastric meat, excluding eggs, increase the most in *Save Yourself* and *Civil Society to the Rescue?*, but the focus and investment in the monogastric industry in *Cash, Control, Calories* keeps the price from increasing as is seen in the other scenarios. In the *Self-Determination* scenario, the price of monogastrics increases only slightly (~2%) by 2050 despite the tripling of per capita demand, in part due to improvements in crop yields that provides more feed as well as keeping producer input costs low. For all scenarios, climate change increases prices for monogastric meat, due to increases in prices for feed products.

Despite the large share of production in region, in all the scenarios by 2050, cassava becomes imported, with imports equaling between 12% and 18% the production in the region (in GLOBIOM). In IMPACT cassava is exported in *Self-Determination* only, in the other scenarios the region imports quantities as large as 13% to 25% of the regional production. Cassava is primarily seen as a staple food crop and is consumed less as incomes rise, while it is also utilized as livestock feed making it an important crop due to a growing demand for meat products from rising income (Figure G3). It is no surprise then that among the scenarios the share of cassava used for livestock feed accounts for 68% of the demand for *Self-Determination*, due to growing incomes and meat demand, and only 26% for *Save Yourself* (in GLOBIOM).

Variability in GLOBIOM Prices by Scenario (over time and under climate change)

Figures G1a and Figures G1b presents the percent deviation of the average of all regional crop prices (weighted by total demand) from the average of all crop prices in 2010 for all scenarios under climate change from GLOBIOM. Under climate change in 86% of the cases there is an increase in the average crop price compared to 2010, whereas under no climate change, only 70% of the cases see prices higher than the 2010 levels. When we examine the differences in prices between the CCAFS scenarios, we see that the average crop price is higher than the 2010 levels for all years and climate change scenarios in *Civil Society to the Rescue?* And *Save Yourself*. The average crop price for 60% of the cases in *Self-Determination* was lower than the 2010. *Save Yourself* has the largest deviations

(and highest variance) from the 2010 prices both under climate change and under no climate change (+45%/+3%).

When we examine the variability of prices over all climate scenarios and decades by crop, we can see that millet and groundnuts have the largest deviations in crop prices, across all scenarios but especially under Save Yourself (Figure G1a and Figure G4). The variability in prices is largest in *Save Yourself* for the majority of major crops (73%).

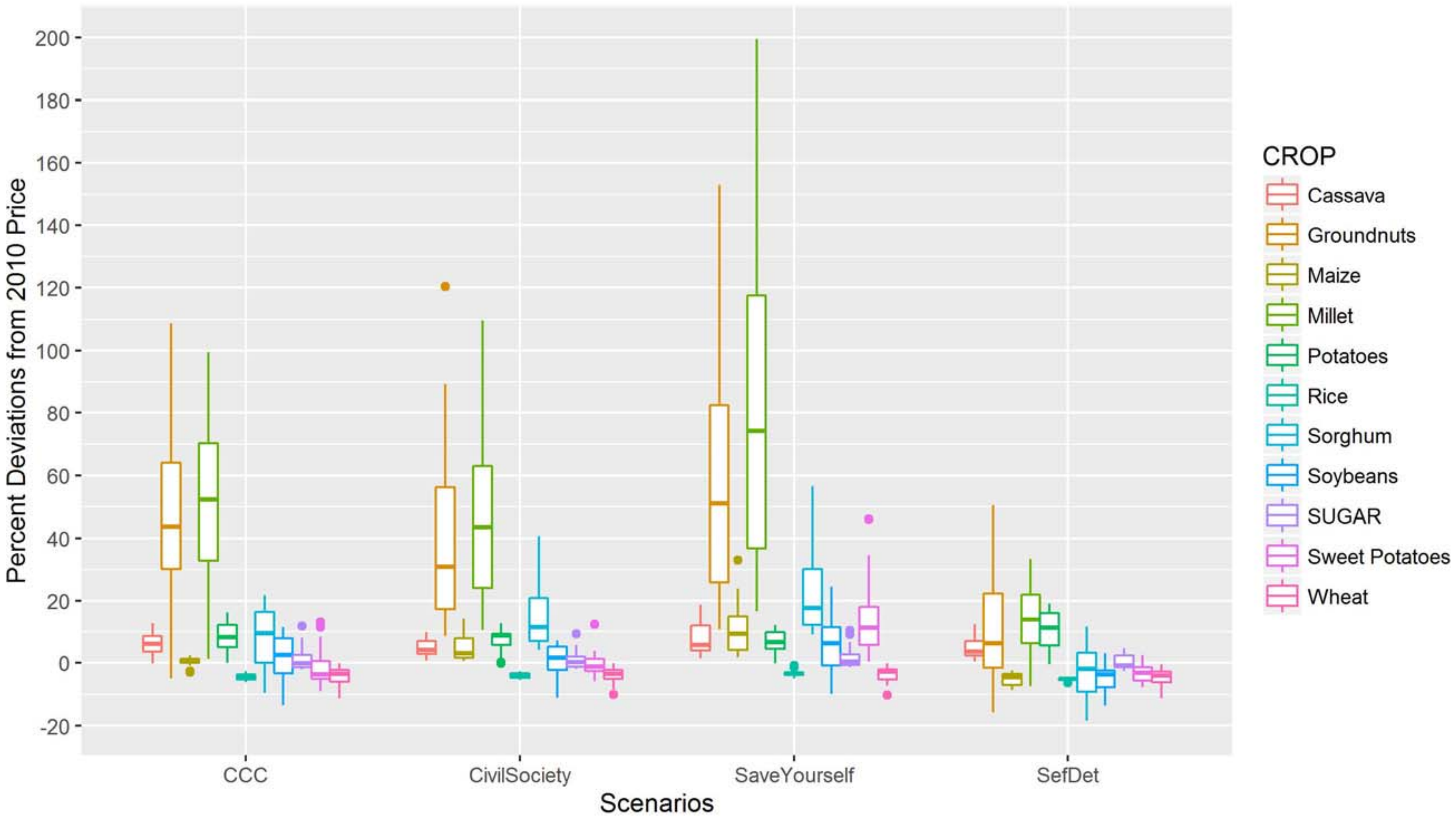

Figure G1a. Deviations from the 2010 indexed price for crops for all GCM simulations and decades from GLOBIOM

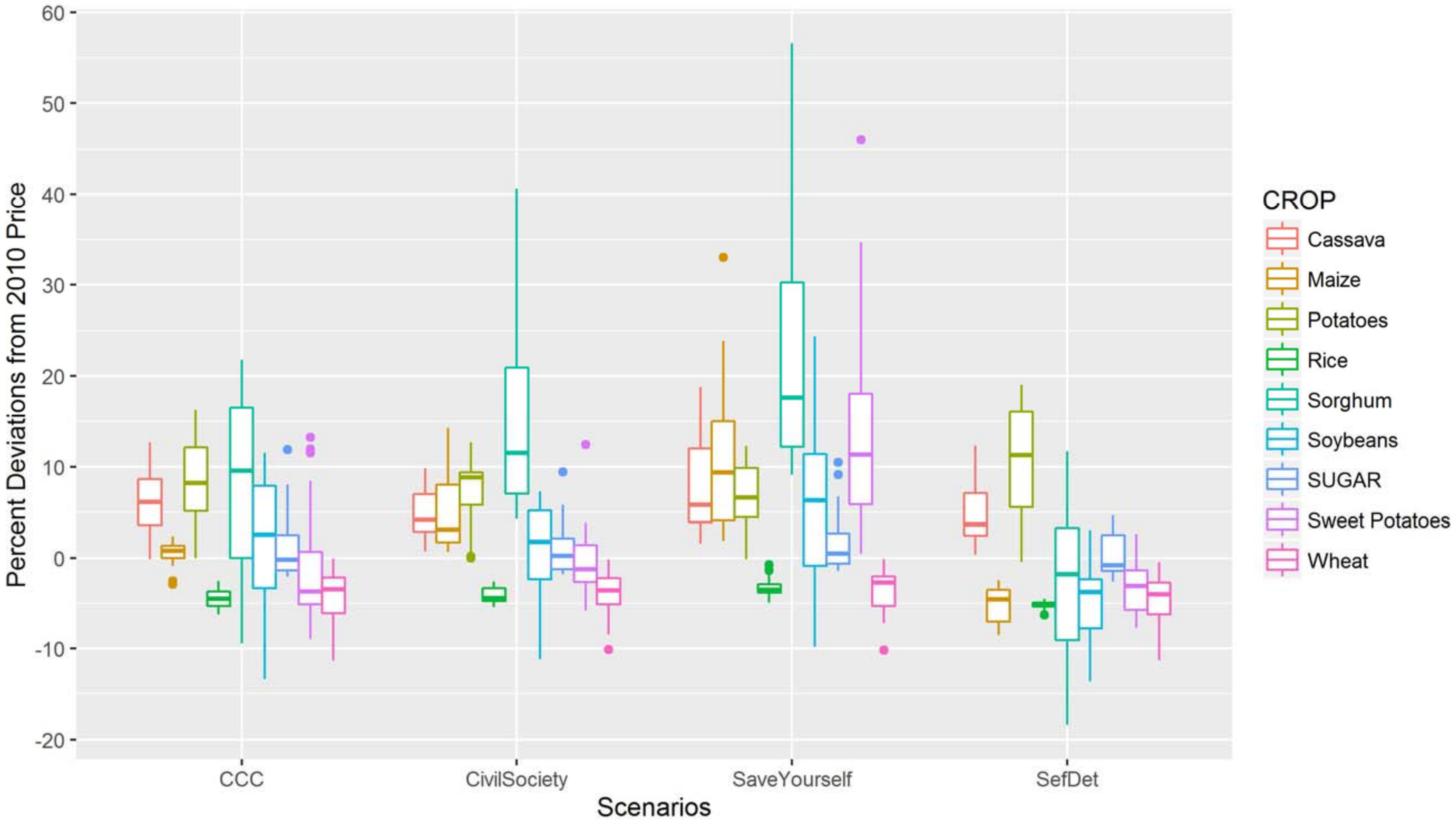

Figure G1b. Deviations from the 2010 indexed price for crops for all GCM simulations and decades from GLOBIOM (without millet and groundnuts)

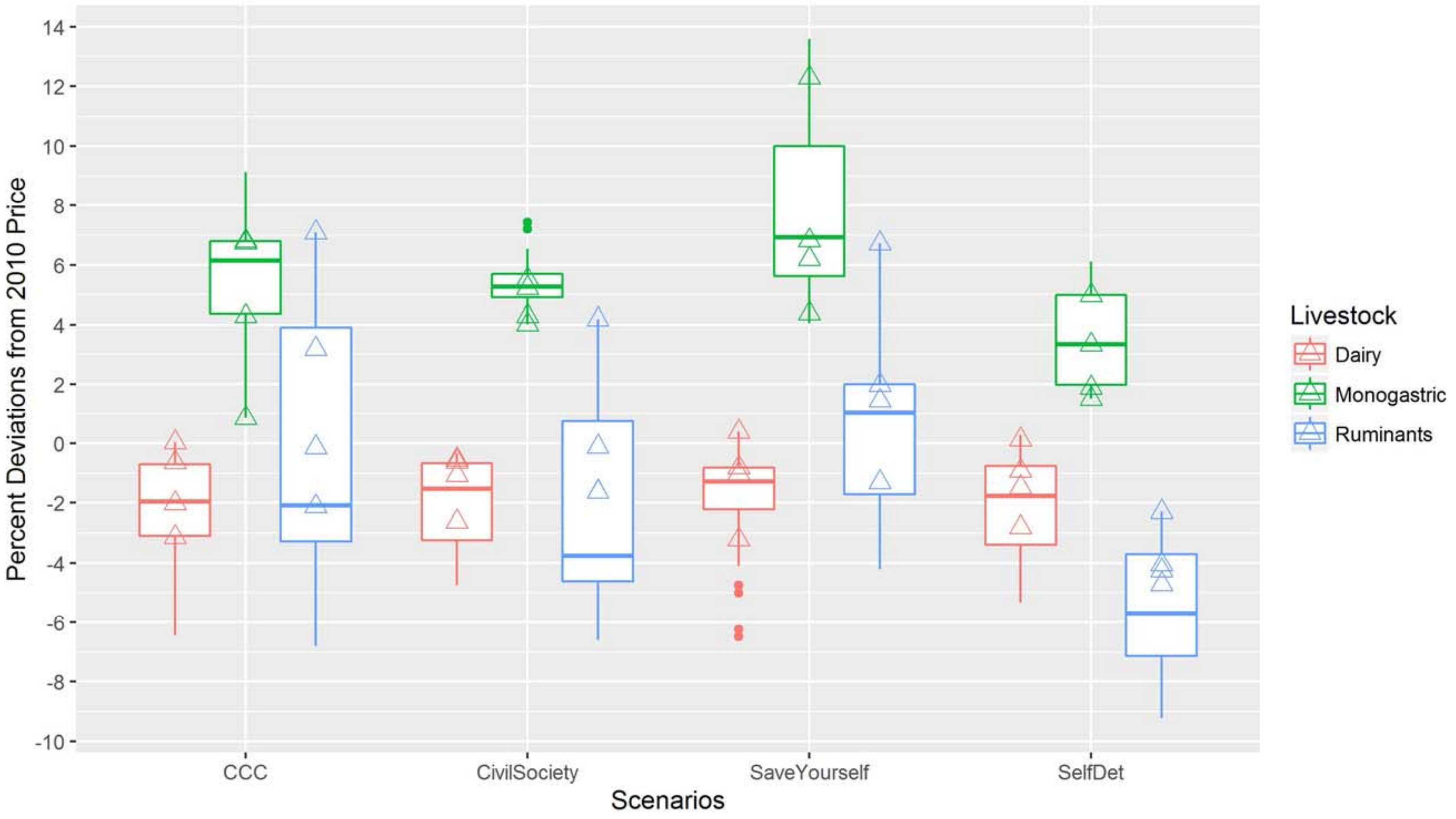

Figure G2. Percent deviations from the 2010 indexed price for livestock products for all GCM simulations and decades

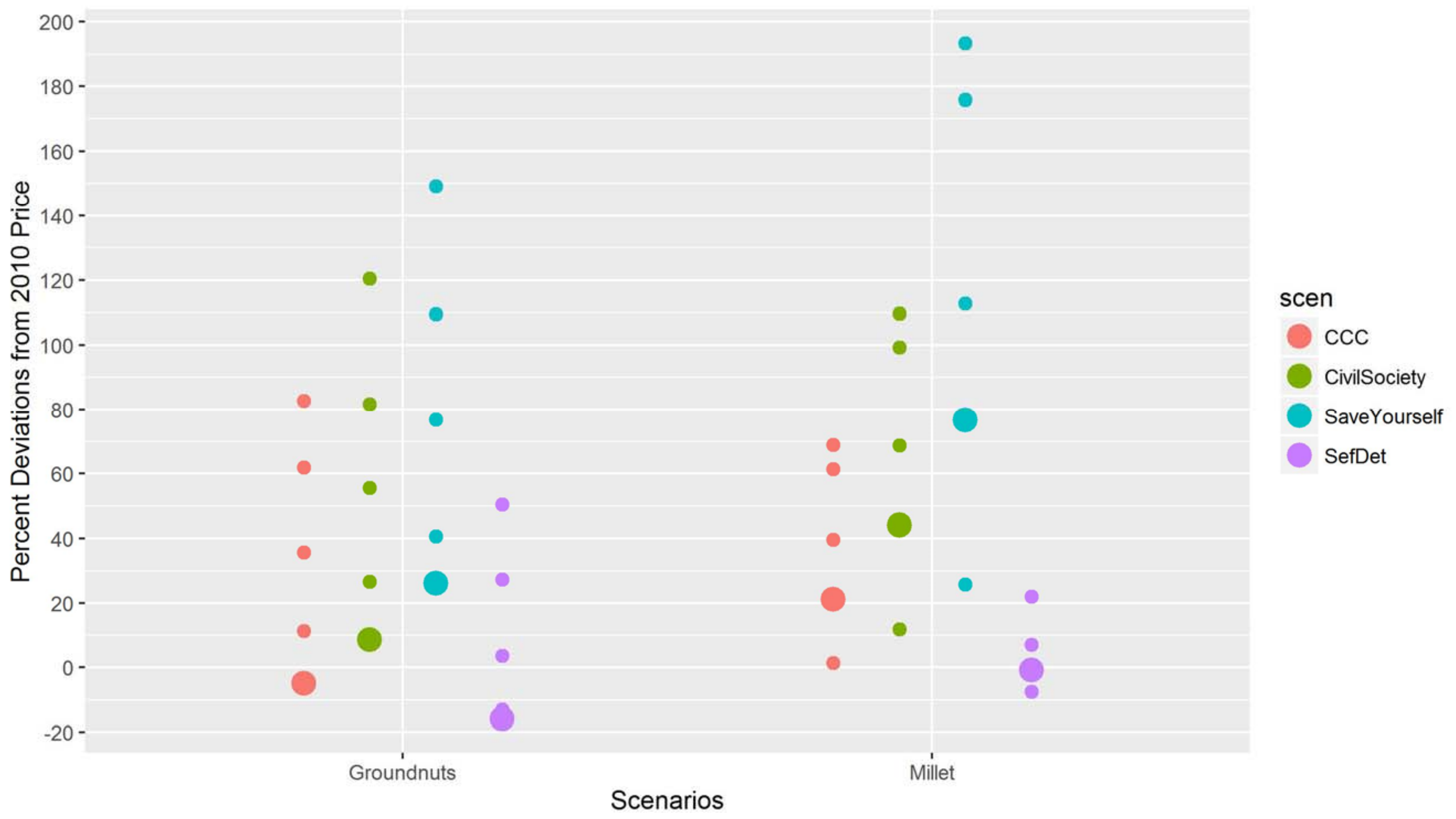

Figure G3. Deviations from the 2010 indexed price for millet and groundnuts over all GCM simulations in 2050 from GLOBIOM

Note: Each large circle represents the deviation in price for the no climate change 2050 future. Each color represents the different CCAFS scenarios.

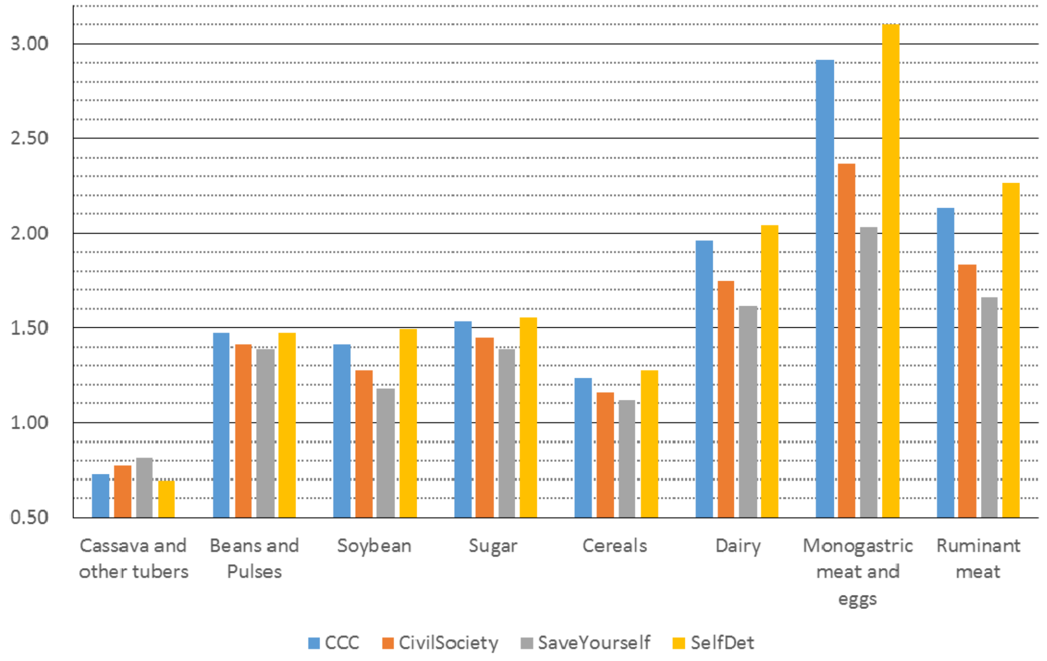

Figure G3. Indexed per capita demand for crop and livestock products (2010=1) for CCAFS scenarios in 2050.

H. Development outside West Africa

In all the scenarios by 2050, socioeconomic growth in the rest of the world increases thus inducing increased demand by 42% for crop products and 65% for livestock products. In 2010, South Asia, China, and South America were the largest consumers of crop products, but by 2050, South Asia and all of Africa will see the largest growth in demand for both crop and livestock products. South America (including Brazil) will become the largest producer for crop and livestock products, followed by South Asia. In terms of food security outside the region, kilocalorie consumption improves in nearly all regions, with India and the rest of South Asia calorie consumption increasing by 15% and 23%. The increase in values for Eastern, Southern, the Congo Basin and the rest of Sub-Saharan Africa will be 31%, 26%, 35%, and 37% by 2050 according to GLOBIOM.

I. Vulnerability and Challenges to adaptation

The indicators of vulnerability factors are categorized according to dimensions outlined from Füssel (2007), which we separate by model assumptions (in grey) and model results (in white). Because GLOBIOM is a partial-equilibrium, economic and land-use model with global coverage we can further examine vulnerability characteristics of the system that stem from internal (or regional) and external (or outside the region) sources.

Table I1. Vulnerability indicators and ranking of indicators CCAFS scenario from low to high vulnerability, with factors categorized according to the dimensions from Füssel (2007)

Sphere and knowledge domain	Indicator	Vulnerability<br>Low			High
Internal Socioeconomic	Food Security	Orange	Blue	Red	Purple
	Diversity of Diet	Orange	Blue	Red	Purple
	Timeline for strategic planning	Orange	Red	Blue	Purple
	Actors Driving Change	Orange	Blue	Red	Purple
	Per capita Income	Orange	Blue	Red	Purple
Internal biophysical	Crop Production	Orange	White	Red / Blue	Purple
	Land Use Change (within West Africa)	Blue	Purple	Red	Orange
	Improved crop and livestock yields	Orange	Blue	Red	Purple
External Socioeconomic	crop prices	Orange	Blue	Red	Purple
	livestock prices	Orange	Blue	Red	Purple
	Net trade of crop products	Orange	Blue	Red	Purple
	Net trade of livestock products	Orange	Blue	Purple	Red
	Regional Integration	Orange	Grey	Red / Blue	Purple
	strength of national government	Orange	Blue	Red	Purple
External biophysical	Land Use Change (outside West Africa)	Orange	Blue	Red	Purple

Cash Control Calories (Blue)	Self Determination (Orange)
Save Yourself (Purple)	Civil Society to the Rescue? (Red)

J. Examples of the use of the CCAFS West-Africa scenarios

A primary purpose for the scenarios discussed in this paper is to use them for national and regional policy guidance (Vervoort et al., 2014). Using a scenario-guided approach, a close collaboration with decision-makers results in the design of a process in which the regional scenarios are downscaled to the national level and to the concerns of a specific plan or policy. These policy guidance examples show that the scenarios bridge global, regional, and national (even sub-national) levels while at the same time linking research on contextual changes in West Africa directly with policy development. This process aims to be inclusive, involving state and non-state actors, including those responsible for the policy and those who are most likely to be affected by it. The scenarios focus strongly on actor interactions and priorities making them both strategically relevant to decision-makers, and imaginable at national and sub-national levels.

The future scenarios offer multiple, challenging contexts in which to test draft plans and policies, providing recommendations for improved strategies which are then integrated. Here, we describe examples where the CCAFS West Africa scenarios were put use to guide the formulation of policies and plans.

- In Burkina Faso, members of the public and private sector and local experts gathered in July 2015 to use the CCAFS West Africa scenarios to review the country's National Plan for the Rural Sector for Burkina Faso (PNSR) in order to formulate a new, more robust PNSR II. Additionally, research priorities were identified needed to help the plan succeed under each of the scenarios. The quantified CCAFS scenarios were useful for adding regional context to the country level scenarios developed by drafters of the PNSR.
- The CCAFS West Africa scenarios have helped guide and inform district- and national level policy processes in Ghana by facilitating an understanding of the factors that may pose challenges to local development such as population growth, urbanization, and government policies.
- In July 2016, Ghanaian state and non-state actors from both the national and district levels met in Dodowa, Greater Accra Region, to review the Ghana National Livestock Policy. The CCAFS West Africa scenarios were downscaled to the national level of Ghana as well to the district level. Maps were used to indicate interactions between the national and district levels as well as international dynamics in each scenario. Using this multi-level angle, the National Livestock Policy was reviewed, leading to recommendations taking into account cross-level interactions and sub-national differences.
- Informed by these national-level processes, a collaboration with ECOWAS and other research partners has been set up to use the regional scenarios for ECOWAS-level priority setting,

notably with the current process of new reshape of the ECOWAS common agricultural policy to meet the new challenges facing West Africa agriculture (ECOWAP+10).

References

Dellink, R., Chateau, J., Lanzi, E., Magné, B., 2015. Long-term economic growth projections in the Shared Socioeconomic Pathways. Glob. Environ. Chang. doi:10.1016/j.gloenvcha.2015.06.004

Fanta, E., Shaw, T.M., Tang, V.T., 2013. Comparative Regionalisms for Development in the 21st Century: Insights from the Global South.

Füssel, H.M., 2007. Vulnerability: A generally applicable conceptual framework for climate change research. Glob. Environ. Chang. 17, 155–167. doi:10.1016/j.gloenvcha.2006.05.002

Hachigonta, S., Nelson, G.C., Thomas, T.S., Sibanda, L.M. (Eds.), 2013. Southern African agriculture and climate change A comprehensive analysis. Washington D.C. doi:10.2499/9780896292086

Havlík, P., Leclère, D., Valin, H., Herrero, M., Schmid, E., Jean-Francois Soussana, C.M., Obersteiner, M., 2015. Climate change and food systems: global assessments and implications for food security and trade, in: Elbehri, A. (Ed.), . Food Agriculture Organization of the United Nations (FAO), pp. 176–208.

Havlík, P., Schneider, U. a., Schmid, E., Böttcher, H., Fritz, S., Skalský, R., Aoki, K., Cara, S. De, Kindermann, G., Kraxner, F., Leduc, S., McCallum, I., Mosnier, A., Sauer, T., Obersteiner, M., 2011. Global land-use implications of first and second generation biofuel targets. Energy Policy 39, 5690–5702. doi:10.1016/j.enpol.2010.03.030

Havlík, P., Valin, H., Herrero, M., Obersteiner, M., Schmid, E., Rufino, M.C., Mosnier, A., Thornton, P.K., Böttcher, H., Conant, R.T., Frank, S., Fritz, S., Fuss, S., Kraxner, F., Notenbaert, A., 2014. Climate change mitigation through livestock system transitions. Proc. Natl. Acad. Sci. U. S. A. 111, 3709–14. doi:10.1073/pnas.1308044111

Hillocks, R.J., 2002. Cassava in Africa., in: Cassava: Biology, Production and Utilization. pp. 41–54. doi:10.1079/9780851995243.0041

Hoogenboom, G., Jones, J.W., Wilkens, P.W., Porter, C.H., Boote, K.J., Hunt, L.A., Singh, U., Lizaso, J.L., White, J.W., Uryasev, O., Royce, F.S., Ogoshi, R., Gijsman, A.J., Tsuji, G.Y., Koo, J., 2012. Decision Support System for Agrotechnology Transfer (DSSAT).

Jalloh, A., Nelson, G.C., Thomas, T.S., Zougmoré, R., Roy-Macauley, H. (Eds.), 2013. West African Agriculture and Climate Change: A Comprehensive Analysis. International Food Policy Research Institute, Washington, D.C. doi:http://dx.doi.org/10.2499/9780896292048

Jones, J.W., Hoogenboom, G., Porter, C.H., Boote, K.J., Batchelor, W.D., Hunt, L. a., Wilkens, P.W., Singh, U., Gijsman, a. J., Ritchie, J.T., 2003. The DSSAT cropping system model, European Journal of Agronomy. doi:10.1016/S1161-0301(02)00107-7

Kc, S., Lutz, W., 2014. The human core of the shared socioeconomic pathways: Population scenarios by age, sex and level of education for all countries to 2100. Glob. Environ. Chang. doi:10.1016/j.gloenvcha.2014.06.004

Leclère, D., Havlík, P., Fuss, S., Schmid, E., Mosnier, A., Walsh, B., Valin, H., Herrero, M., Khabarov, N., Obersteiner, M., 2014. Climate change induced transformations of agricultural systems: insights from a global model. Environ. Res. Lett. 9, 124018. doi:10.1088/1748-9326/9/12/124018

Mosnier, A., Obersteiner, M., Havlík, P., Schmid, E., Khabarov, N., Westphal, M., Valin, H., Frank, S., Albrecht, F., 2014. Global food markets, trade and the cost of climate change adaptation. Food Secur. 6, 29–44. doi:10.1007/s12571-013-0319-z

Nelson, G.C., Rosegrant, M.W., Palazzo, A., Gray, I., Ingersoll, C., Robertson, R., Tokgoz, S., Zhu, T., 2010. Food Security, Farming, and Climate Change to 2050: Scenarios, Results, Policy Options, Research reports IFPRI. International Food Policy Research Institute. doi:10.2499/9780896291867

Nelson, G.C., Rosegrant, M.W., Palazzo, A., Gray, I., Ingersoll, C., Robertson, R., Tokgoz, S., Zhu, T., 2010. Gerald C. Nelson, Mark W. Rosegrant, Amanda Palazzo, Ian Gray, Christina Ingersoll, Richard Robertson, Simla Tokgoz, Tingju Zhu, Timothy B. Sulser, Claudia Ringler, Siwa Msangi, and Liangzhi You, Research reports IFPRI. doi:10.2499/9780896291867

Nelson, G.C., Valin, H., Sands, R.D., Havlík, P., Ahammad, H., Deryng, D., Elliott, J., Fujimori, S., Hasegawa, T., Heyhoe, E., Kyle, P., Von Lampe, M., Lotze-Campen, H., Mason d'Croz, D., van Meijl, H., van der Mensbrugghe, D., Müller, C., Popp, A., Robertson, R., Robinson, S., Schmid, E., Schmitz, C., Tabeau, A., Willenbockel, D., 2014a. Climate change effects on agriculture: economic responses to biophysical shocks. Proc. Natl. Acad. Sci. U. S. A. 111, 3274–9. doi:10.1073/pnas.1222465110

Nelson, G.C., van der Mensbrugghe, D., Ahammad, H., Blanc, E., Calvin, K., Hasegawa, T., Havlik, P., Heyhoe, E., Kyle, P., Lotze-Campen, H., von Lampe, M., Mason d'Croz, D., van Meijl, H., Müller, C., Reilly, J., Robertson, R., Sands, R.D., Schmitz, C., Tabeau, A., Takahashi, K., Valin, H., Willenbockel, D., 2014b. Agriculture and climate change in global scenarios: why don't the models agree. Agric. Econ. 45, 85–101. doi:10.1111/agec.12091

O'Neill, B.C., Kriegler, E., Ebi, K.L., Kemp-Benedict, E., Riahi, K., Rothman, D.S., van Ruijven, B.J., van Vuuren, D.P., Birkmann, J., Kok, K., Levy, M., Solecki, W., 2015. The roads ahead: Narratives for shared socioeconomic pathways describing world futures in the 21st century. Glob. Environ. Chang. 1–48. doi:10.1016/j.gloenvcha.2015.01.004

Palazzo, A., Rutting, L., Vervoort, M., Havlik, P., Jalloh, A., Aubee, E., Helfgott, A.E.S., Ericksen, P.J., Abdoulaye, S., Bayala, J., Kadi, H.A.K., Sibiry, P.C., Thornton, P.K., 2016. The future of food security, environments and livelihoods in Western Africa: Four socio-economic scenarios (No. 130), CGIAR Research Program on Climate Change, Agriculture and Food Security (CCAFS) Working Paper. Copenhagen, Denmark.

Palazzo, A., Vervoort, J., Havlik, P., Mason-D 'croz, D., Islam, S., 2014. Simulating stakeholder- driven food and climate scenarios for policy development in Africa, Asia and Latin America: A multi-regional synthesis CGIAR Research Program on Climate Change, Agriculture and Food Security (CCAFS) (No. 109), CGIAR Research Program on Climate Change, Agriculture and Food Security (CCAFS) Working Paper. Copenhagen, Denmark.

Robinson, S., Mason-D'Croz, D., Islam, S., Sulser, T.B., Robertson, R., Zhu, T., Gueneau, A., Pitois, G., Rosegrant, M., Technology, E. and P., 2015. The International Model for Policy Analysis of Agricultural Commodities and Trade (IMPACT) Model Description for Version 3 (No. 1483), IFPRI Discussion Paper. Washington D.C.

Sultan, B., Roudier, P., Quirion, P., Alhassane, a, Muller, B., Dingkuhn, M., Ciais, P., Guimberteau, M., Traore, S., Baron, C., 2013. Assessing climate change impacts on sorghum and millet yields in the Sudanian and Sahelian savannas of West Africa. Environ. Res. Lett. 8, 14040. doi:10.1088/1748-9326/8/1/014040

von Lampe, M., Willenbockel, D., Ahammad, H., Blanc, E., Cai, Y., Calvin, K., Fujimori, S., Hasegawa, T., Havlik, P., Heyhoe, E., Kyle, P., Lotze-Campen, H., Mason d'Croz, D., Nelson, G.C., Sands, R.D., Schmitz, C., Tabeau, A., Valin, H., van der Mensbrugghe, D., van Meijl, H., 2014. Why do global long-term scenarios for agriculture differ? An overview of the AgMIP Global Economic Model Intercomparison. Agric. Econ. 45, 3–20. doi:10.1111/agec.12086

Waithaka, M., Nelson, G.C., Thomas, T.S., Kyotalimye, M. (Eds.), 2013. East African agriculture and climate change A comprehensive analysis. doi:10.2499/9780896292055

Wheeler, T., Reynolds, C., 2012. Predicting the risks from climate change to forage and crop production for animal feed. Anim. Front. 3, 36–41. doi:10.2527/af.2013-0006
